# MRI-free processing of tau PET images for early detection

**DOI:** 10.1162/imag_a_00369

**Published:** 2024-11-13

**Authors:** Mackenzie L. Carlson, Viktorija Smith, Emily Johns, Christina B. Young, Hillary Vossler, Tyler Ward, Theresa M. Harrison, Duygu Tosun, Timothy Hohman, Susan M. Landau, Elizabeth C. Mormino

**Affiliations:** Departments of Neurology and Neurological Sciences, Stanford University, Stanford, CA, United States; Helen Wills Neuroscience Institute, UC Berkeley, Berkeley, CA, United States; Department of Radiology and Biomedical Imaging, University of California San Francisco, San Francisco, CA, United States; Department of Neurology, Vanderbilt University Medical Center, Nashville, TN, United States; Wu Tsai Neuroscience Institute, Stanford, CA, United States

**Keywords:** PET imaging, image processing, tau, Alzheimer’s Disease

## Abstract

Tau positron emission tomography (PET) imaging in Alzheimer’s Disease (AD) is becoming increasingly common to assess*in vivo*tau burden. MR images are often acquired to assist with processing of PET data, including for region-of-interest definitions in native space and for normalization to template space. However, in the real-world setting, corresponding MRIs may not be available and PET processing may require MRI-free pipelines. This is particularly important and challenging as the field moves towards early detection among clinically unimpaired (CU) individuals where changes in tau PET signal are expected to be subtle. We used two independent [^18^F]Flortaucipir tau PET datasets to evaluate whether MRI-free PET processing can detect subtle tau PET uptake differences in Amyloid+ (A+) CU individuals (preclinical AD) versus A-. Standardized Uptake Value Ratios (SUVRs) from MRI-free compared to MRI-based methods were evaluated using linear regression and linear mixed-effects regression models. Effect size differences between A+/- CU groups in MRI-free processed cross-sectional and longitudinal tau PET SUVRs were compared to differences quantified through MRI-based processing. Regional MRI-free SUVRs were highly correlated with MRI-based SUVRs within CU individuals (average ICC = 0.90 for ADNI CU and 0.81 for A4 CU). MRI-free and MRI-based pipelines resulted in similar estimates of cross-sectional and longitudinal differences between A- and A+ CU, even in early focal regions within the medial temporal lobe.

## 1 Introduction

Positron emission tomography (PET) imaging in Alzheimer’s Disease (AD) is becoming increasingly widespread to assess*in vivo*β-amyloid and tau pathology burden. In the context of clinical trials, accurate quantification of tau burden is critical to determining treatment efficacy and monitoring treatment response (Cummings et al., 2023;Mintun et al., 2021;Ossenkoppele, van der Kant, et al., 2022;Sims et al., 2023;Van Dyck et al., 2023;Verger et al., 2023). In the research setting, quantitative approaches to tau PET imaging often rely on an accompanying anatomical MRI scans. Given the poor spatial resolution of PET, MRI can be used to define regions of interest that are applied to the co-registered PET data or PET template to extract regional PET uptake values. However, in both the research and real-world clinical setting, corresponding MRIs may not be available and alternative MRI-free PET processing may be required for quantification of tau PET burden.

MRI-free analysis pipelines have already been developed for evaluating amyloid PET (Bourgeat et al., 2015;Doré et al., 2019;Edison et al., 2013;Iaccarino et al., 2022;Pegueroles et al., 2021;Ward et al., 2023) and, to a lesser extent, tau PET (Fleisher et al., 2020;Landau et al., 2023). In a study byLandau et al. (2023), the association between MRI-free and MRI-based processing pipelines was R^2^= 0.95 for both cross-sectional [^18^F]Florbetapir and [^18^F]Florbetaben amyloid PET. Similarly, a study byIaccarino et al. (2022)showed high consistency between MRI-free and MRI-based methods for determining amyloid status (86–94% agreement). However, it remains unclear whether MRI-free pipelines for tau PET may result in less precise estimates, given the regional nature of tau deposition. Initial characterization of MRI-free pipelines across the AD clinical spectrum for tau PET has been promising, withLandau et al. (2023)showing high correspondence ranging from R^2^= 0.88 to R^2^= 0.97 in entorhinal and composite temporal regions with cross-sectional [^18^F]Flortaucipir (FTP) PET data. However, characterizing the ability of MRI-free pipelines to detect subtle changes within cognitively unimpaired (CU) and preclinical AD patients has not specifically been evaluated.

Given an increasing interest in early detection of AD pathology in CU individuals (Ossenkoppele, Pichet Binette, et al., 2022;Rafii et al., 2023;Sperling et al., 2014,^2020^), it is important to establish whether tau PET MRI-free methods provide consistent results when applied during the early stages of pathological accumulation. The goal of this work was, therefore, to evaluate the sensitivity of MRI-free processing applied to tau PET imaging in the context of subtle elevations among CU individuals. Furthermore, it is important to evaluate MRI-free processing in longitudinal data in order to assess the ability to monitor early tau accumulation.

## 2 Materials and Methods

### 2.1 Tau PET datasets

This study includes cross-sectional analyses of two independent [^18^F]FTP datasets: the Alzheimer’s Disease Neuroimaging Initiative (ADNI) (https://adni.loni.usc.edu) (921 unique participants) (Jagust et al., 2010,^2015^) and the Anti-Amyloid Treatment in Asymptomatic Alzheimer’s study (A4) (447 unique participants, single timepoint) (Sperling et al., 2014;Young et al., 2022). We additionally performed longitudinal analysis on a subset of the cross-sectional ADNI dataset, including 193 ADNI participants with 3 or more [^18^F]FTP scans (3.3 ± 0.6 scans over 3.2 ± 1.2 years) (Table 1).

**Table 1.** Summary of all cross-sectional image data across both cohorts and the longitudinal subset of ADNI data, including only participants with three or more tau PET scans.

Demographics		Cross-Sectional		Longitudinal (3+ Scans)
		ADNI Unique participants at baseline	A4	ADNI Unique participants at baseline
Age m (SD)		73.4 (7.98)	71.8 (4.84)	73.3 (7.87)
Sex n (%)	Male	442 (48.0%)	257 (57.5%)	92 (47.7%)
	Female	479 (52.0%)	190 (42.5%)	101 (52.3%)
CU	A–	332 (36.0%)	68 (15.2%)	50 (25.4%)
	A+	187 (20.3%)	379 (84.8%)	52 (26.6%)
MCI	A–	148 (16.1%)	-	26 (13.6%)
	A+	150 (16.3%)	-	42 (23.7%)
AD	A–	14 (1.5%)	-	2 (1.1%)
	A+	90 (9.8%)	-	21 (9.6%)
**Total Scans**		**921**	**447**	**193**

The primary goal of ADNI has been to test whether serial MRI, PET, other biological markers, and clinical and neuropsychological assessment can be combined to measure the progression of MCI and early AD. For up-to-date information, seewww.adni-info.org. The A4 study is a secondary prevention trial that focused on participants with preclinical AD (Sperling et al., 2014). All participants provided written informed consent, and these studies were approved by local institutional review boards.

### 2.2 Clinical diagnosis

Participants were grouped by clinical diagnosis (CU = clinically unimpaired, MCI = mild cognitive impairment, AD dementia) at the visit closest in time to the baseline tau PET scan. ADNI diagnoses were based on the following criteria: CU were free of memory complaints, Clinical Dementia Rating score (CDR) = 0, and Mini-Mental State Examination score (MMSE) = 24–30; MCI had a subjective memory concern, MMSE = 24–30, CDR = 0.5, and did not meet criteria for dementia; AD dementia had a subjective memory concern, CDR = 0.5–1, MMSE = 20–26, and met NINCDS/ADRDA criteria for probable AD (R. C. Petersen et al., 2010). ADNI participants with clinical diagnoses of non-AD neurodegenerative diseases were excluded for the current analyses. All A4 participants were CU (CDR = 0, MMSE = 25–30, and Logical Memory Delayed Recall score = 6–18) (Sperling et al., 2014).

### 2.3 Amyloid PET acquisition and processing

Amyloid PET was used for determining amyloid status (positivity or negativity) for each participant at baseline. Amyloid PET was not used for processing the tau PET data with the MRI-Free pipeline. For ADNI, [^18^F]Florbetapir (N = 565/921 scans) was acquired between 50 and 70 minutes post-injection, [^18^F]Florbetaben (N = 354/921 scans) was acquired between 90 and 110 minutes post-injection, and [^11^C]Pittsburgh Compound-B (N = 2/921 scans) was acquired between 50 and 70 minutes post-injection. For A4, all 447 participants underwent [^18^F]Florbetapir imaging, with data collected 50–70 minutes post-injection.

ADNI and A4 amyloid PET data were processed locally at Stanford using an MRI-Free pipeline described byLandau et al. (2023). Five-minute amyloid PET frame data were motion corrected and summed. These summed files were used to linearly co-register PET data to the MNI-152 T1 1 mm template using SPM12 (www.fil.ion.ucl.ac.uk/spm). Co-registered PET data were then nonlinearly warped to a generic amyloid tracer template. Intensity values were extracted from a global target region from the GAAIN atlas (Klunk et al., 2015) and normalized to the GAAIN whole cerebellum reference region to create Standardized Uptake Values Ratios (SUVRs).

SUVRs were converted to CL using standard approaches (Klunk et al., 2015;Navitsky et al., 2018;Rowe et al., 2017) (Supplementary Methods 1). An SUVR value of 1.17 (CL = 12) was used to define positivity for [^18^F]Florbetapir (Supplementary Fig. 1), 1.11 (centiloids (CL) = 18) was used to define positivity for [^18^F]Florbetaben (Supplementary Fig. 2), and 1.13 (CL = 9) was used to define positivity for [^11^C]Pittsburgh Compound-B (Supplementary Figs. 3and 5;Supplementary Table 1). Methods for determining SUVR cutoffs are described inSupplementary Methods 1.

### 2.4 Tau PET acquisitions and preprocessing

For the [^18^F]FTP ADNI dataset, summed images corresponding to 75–105 minutes post-injection were directly downloaded from LONI (using the most processed data from the ADNI PET Core, labeled as “AV1451 Coreg, Avg, Std Img and Vox Siz, Uniform Resolution”). For the [^18^F]FTP A4 dataset, frame data corresponding to 80–110 minutes post-injection were downloaded from LONI, realigned, and summed. Summed images across both datasets were created for processing through both MRI-based and MRI-free pipelines.

### 2.5 Tau PET MRI-based pipeline

As a ground-truth tau PET processing method, we implemented a FreeSurfer MRI-based pipeline currently used in ADNI (Landau et al., 2012;Mormino et al., 2009) for the analysis of A4 tau PET data (Young et al., 2022). ADNI tau PET data were not processed locally since already available with this same pipeline (UCBERKELEYAV1451_8mm_02_17_23_10Jul2023.csv). In brief, each participant’s structural T1-weighted MRI was processed through FreeSurfer v7.1. Each participant’s T1 data were co-registered to the corresponding summed tau PET data, to enable alignment of FreeSurfer aparc+aseg atlas labels (Fischl, 2012) with the PET data. Mean uptake values were extracted from each region, and bilateral volume-weighted SUVRs were calculated using the inferior cerebellar cortex as a reference region, defined by the aparc+aseg region for A4, and overlap between the SUIT inferior cerebellar (Diedrichsen, 2006) and FreeSurfer cerebellum gray matter masks (Baker et al., 2017;Landau et al., 2023;Maass et al., 2017) in ADNI.

### 2.6 Tau PET MRI-free pipeline

Image processing was done using SPM12 (http://www.fil.ion.ucl.ac.uk/spm/) in MATLAB R2020b. Summed PET files were co-registered to a 1 mm isotropic T1-weighted MRI template in Montreal Neurological Institute (MNI)-152 space; parameters used for spatial coregistration are written inSupplementary Methods 2. Then, images were warped to tau PET templates and SUVs were extracted from regions of interest (ROIs) based on the Normalized Probability Desikan-Killiany Atlas (NPDKA), a template space atlas that parallels the Desikan-Killiany atlas typically generated in native space by FreeSurfer (Desikan et al., 2006). The NPDKA was developed by UC Berkeley by warping 200 ADNI A- CU participants native space aparc+aseg labels to template space and then determining ROI-membership for each template space voxel based on the highest probability for that voxel across the 200 warped atlases (Ward et al., 2023). This enabled quantification of analogous ROIs as the MRI-based streams (Fischl et al., 2002;Mormino et al., 2009). SUVR values were computed by normalizing to an NPDKA-derived inferior cerebellum cortex reference region. A pipeline overview is shown inFigure 1.

**Fig. 1.**
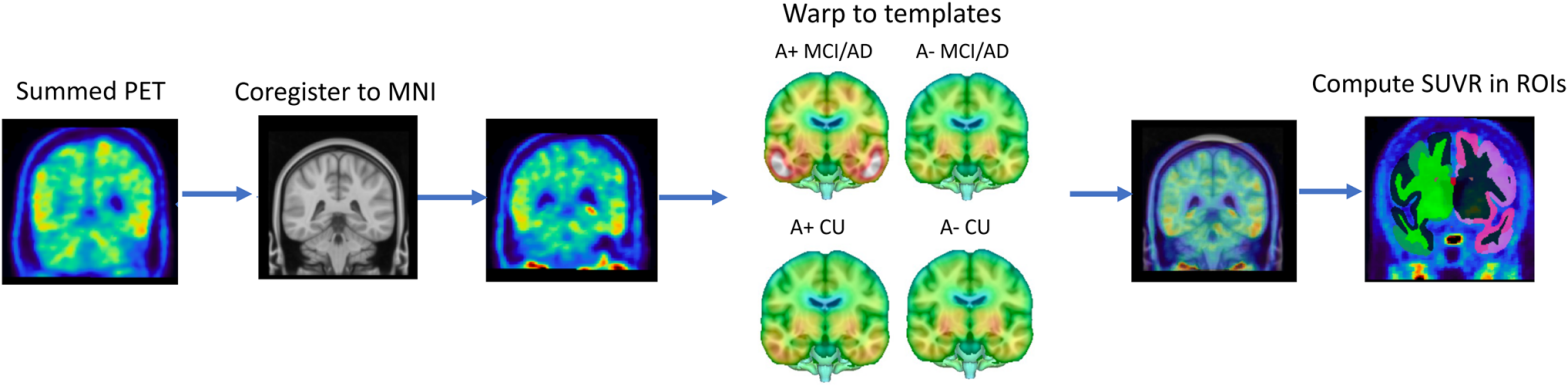
Schematic overview of MRI-free tau PET processing pipeline.

#### 2.6.1 Tau PET template creation

We evaluated two template approaches to guide spatial normalization. First, we used four separate [^18^F]FTP templates simultaneously (“multi-template”), each template being a within-group average of A- CU, A+ CU, A- MCI/AD, and A+ MCI/AD groups (N = 15, randomly selected) to account for differences in binding patterns across the disease spectrum (Johnson et al., 2016;G. C. Petersen et al., 2022) (Supplementary Methods 2;Supplementary Fig. 6). Spatial normalization using a multi-template approach was done in SPM12 using the oldnorm toolbox. Using each individual summed tau image resliced in MNI space as the source image, SPM derives a weighted linear combination across the 4 tau templates that minimizes the residual squared difference between the source image and template combination (Ashburner & Friston, 1999) (see SPM12 manual athttps://www.fil.ion.ucl.ac.uk/spm/doc/spm12_manual.pdf). In the second approach, we used a single merged [^18^F]FTP template, which is the unweighted average of the four separate templates. Then, using the oldnorm toolbox in SPM, spatial normalization for both approaches was completed which iteratively warps the PET image into template space. Default parameters were used and are written inSupplementary Methods 2. All templates were created using ADNI data and applied to both ADNI and A4 cohorts to evaluate these approaches both within cohort (ADNI) and in an external cohort (A4).

**Fig. 2.**
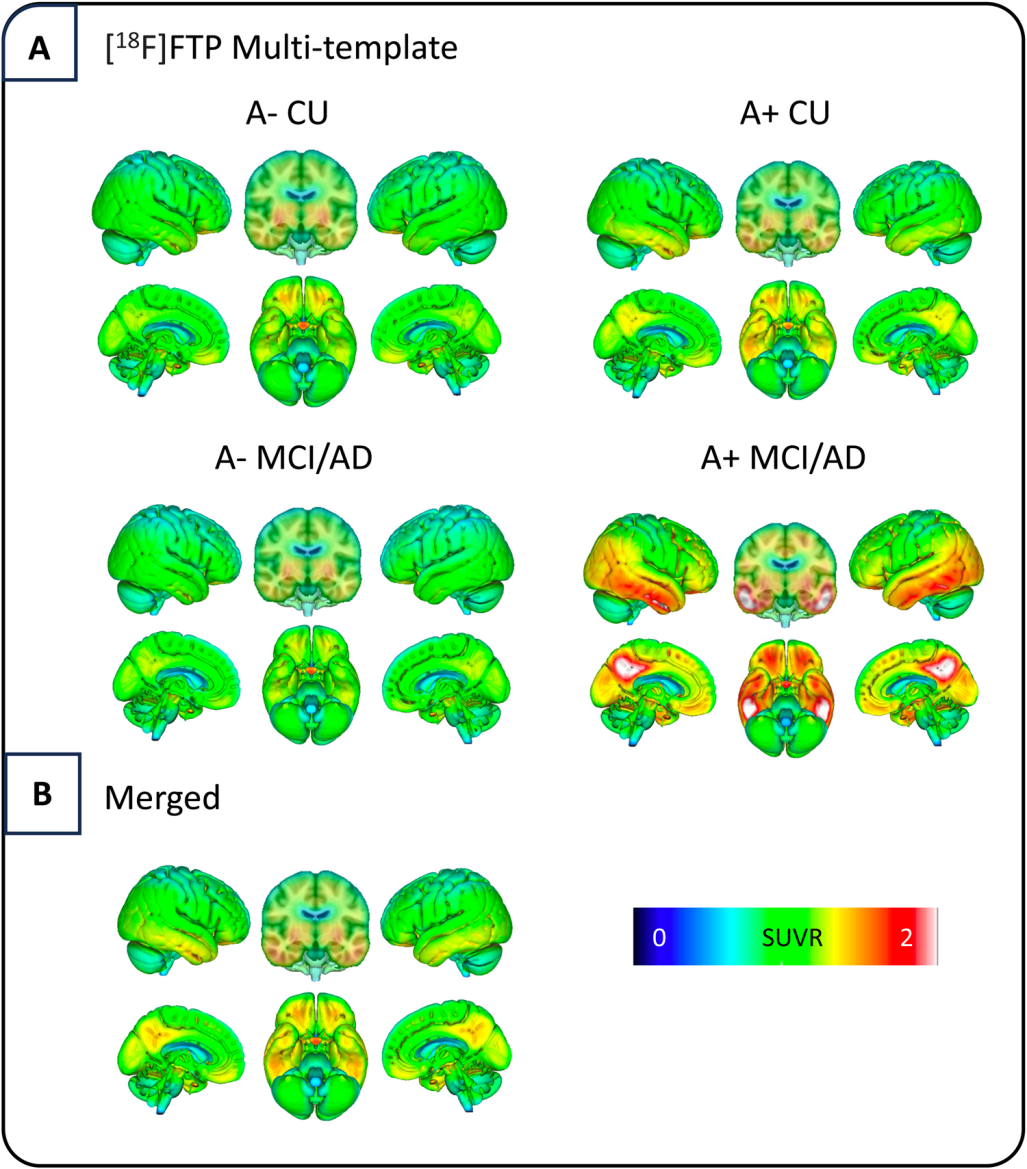
(A) SUVR templates generated using ADNI [^18^F]FTP data in four diagnostic groups. (B) Single [^18^F]FTP SUVR template generated by merging all four of the above templates into a single template image.

To create each template, individual summed PET files and corresponding T1-weighted MRI were co-registered in SPM and non-linearly spatially normalized to a T1-weighted 1 mm isotropic MNI brain atlas (www.fil.ion.ucl.ac.uk/spm). SUVR images were generated from each normalized PET file by dividing the image by the mean intensity value of the NPDKA-derived inferior cerebellar cortex (Desikan et al., 2006). Spatially normalized SUVR images were summed to create each template. Templates were smoothed by an 8 mm^3^voxel kernel (Fig. 2).

#### 2.6.2 ROI selection and SUVR cutoff recommendations

ROIs were examined across three common staging frameworks present in the literature based on known pathologic progression of typical AD (Supplementary Table 2). These ROIs reflect approaches taken in the literature to examine early tau accumulation (Landau et al., 2023), Braak staging like spread (Braak & Braak, 1991;Braak et al., 2006;Pascoal et al., 2020,^2021^;Therriault et al., 2022), and atypical tau patterns throughout cortex (Ossenkoppele et al., 2016;Young et al., 2022). The hippocampus was excluded from composite regions due to potential high off-target binding in the choroid plexus (Baker et al., 2019).

### 2.7 Statistical analysis

All statistical analyses were conducted in R v4.2.2. Linear models and intraclass correlation (ICC, measuring agreement) were used to compare multi-template MRI-free, merged-template MRI-free, and MRI-based cross-sectional SUVRs. All participants were included in the ICC and R^2^analysis, but A- AD were excluded when contrasting across groups due to small sample size. Cross-sectional differences between diagnostic groups (A- CU, A+ CU, A+ MCI, and A+ AD) were assessed using a general linear regression model. Magnitude differences between pipelines were assessed using paired T-tests between MRI-free and MRI-based SUVR in each ROI for each diagnostic group as well as using a linear regression model controlling for diagnosis. Cohen’s D values comparing effect sizes of cross-sectional differences between groups were computed. Linear mixed-effects models were used to examine change in SUVR over time (e.g., SUVR ~ Time x Group + (1 + Time | SubjectID)), with fixed effects of Group x Time and random intercept and slope predicting SUVR. Individual slopes (SUVR/year) were extracted from the model and used for visualization purposes only (plotting MRI-free versus MRI-based extracted slopes). These models were repeated for each ROI. A pipeline interaction term (e.g., SUVR ~ Time*Group + Time*Pipeline + (1 + Time | SubjectID)) was included to determine whether the magnitude of SUVR change varied by pipeline, controlling for diagnosis. Raw P-values are listed or shown by color code in figures.

## 3 Results

**Fig. 3.**
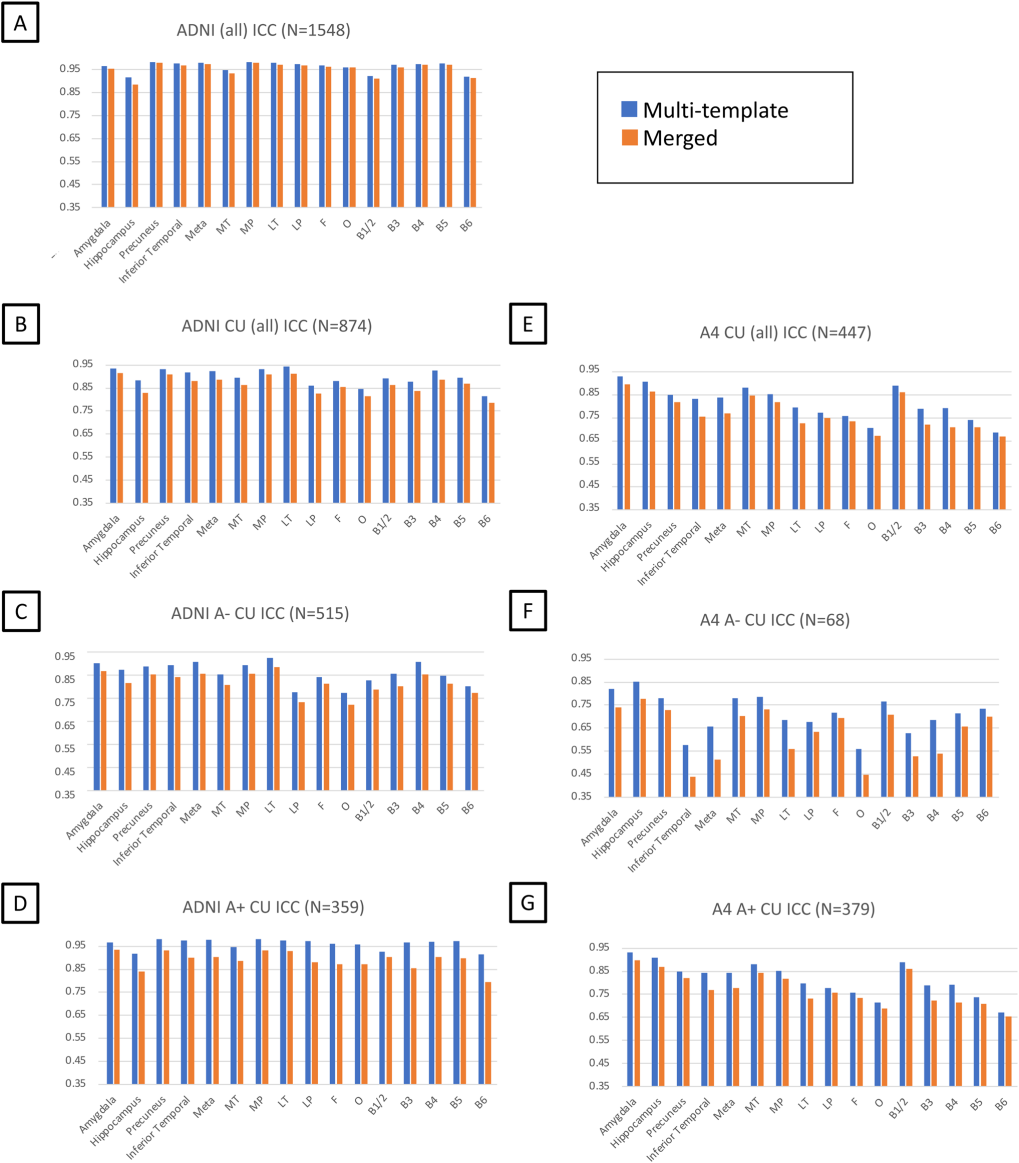
Intraclass correlation between MRI-based and MRI-free SUVRs in tau-relevant ROIs for ADNI (A–D) and A4 (E–G). Meta = meta temporal composite region, MT = medial temporal, MP = medial parietal, LT = lateral temporal, LP = lateral parietal, F = frontal, O = occipital.

Within ADNI, R^2^and ICCs comparing MRI-free approaches (multi-template versus merged FTP template) to MRI-based SUVRs computed demonstrated that the multi-template approach yielded higher ICC (Fig. 3) and R^2^values (Supplementary Fig. 7) across all tau-specific ROIs examined. To assess these pipeline differences for early detection and in an independent cohort, we examined these values within the ADNI CU group only and the A4 CU dataset. We additionally divided the CU group into A- and A+ subgroups to further understand pipeline differences among CU (Fig. 3C, D,F, G;Supplementary Fig. 7C, D, F, G). We found that the multi-template approach resulted in higher R^2^and ICC values than the merged template across these CU subgroup analyses. We also observe higher R^2^and ICC values between MRI-free and MRI-based SUVRs in ADNI compared to A4, which may be because the templates were created using ADNI data. Given the superior correspondence between multi-template MRI-free SUVRs and MRI-based SUVRs, we used the multi-template approach for further evaluation of the MRI-free pipeline in both cohorts.

Although the multi-template MRI-free pipeline was highly correlated with the MRI-Based pipeline (Figs. 3and4;Supplementary Fig. 7), a mean shift was present for all regions such that the MRI-free pipeline generated slightly higher SUVRs (Supplementary Table 3;Fig. 5). This mean shift was confirmed using both T-tests and linear regression to contrast MRI-free and MRI-based SUVRs (Supplementary Table 3). Overall, the MRI-free pipeline produced higher values ranging from 0.009 to 0.054 SUVR units.

**Fig. 4.**
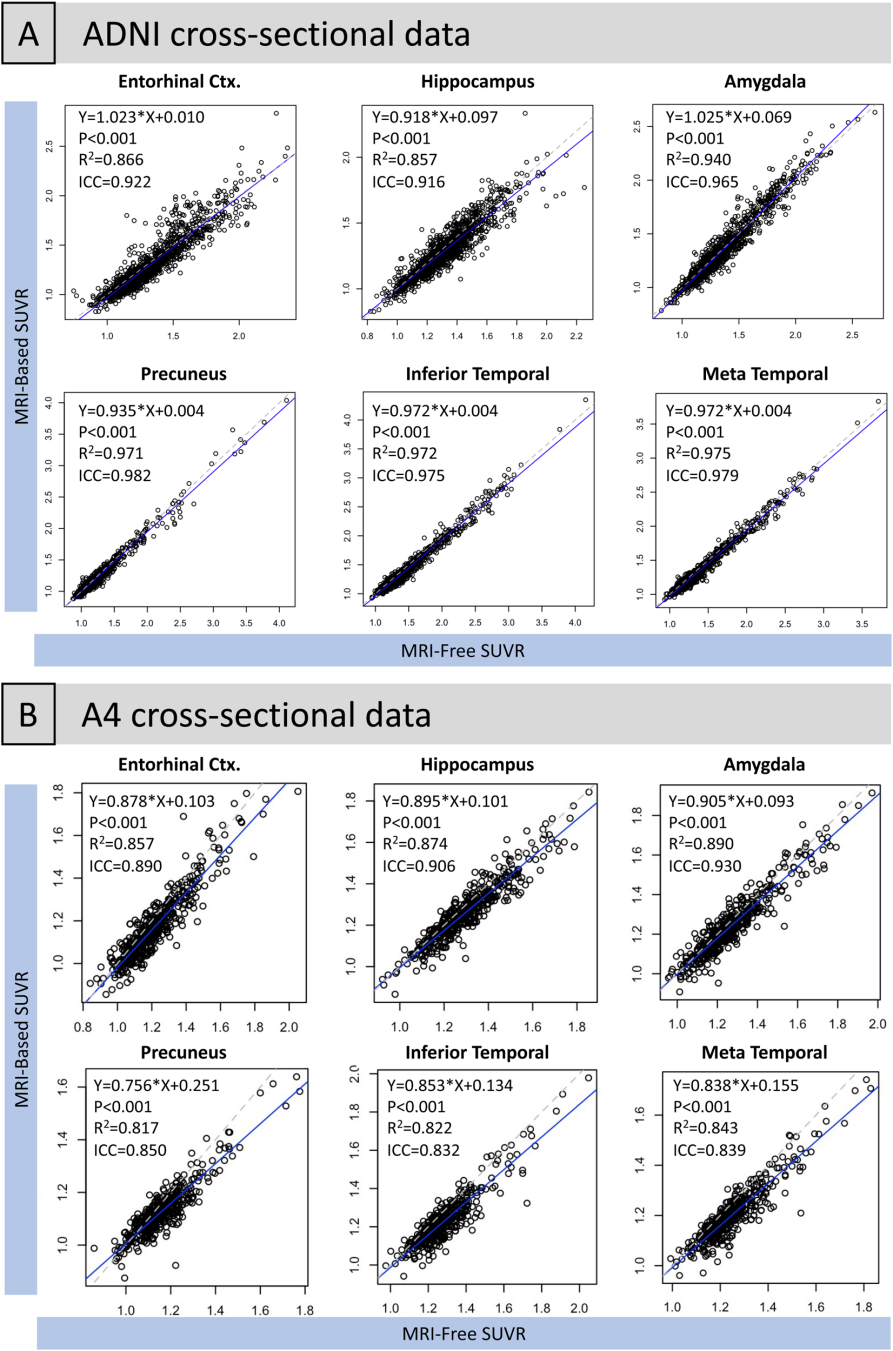
Association between multi-template MRI-free and MRI-based SUVR in regions of early tau accumulation in the entire cohort of (A) ADNI and (B) A4 data. Plots showing the association between the single merged-template MRI-free and MRI-based SUVR regions of early tau are shown inSupplementary Figure 8.

**Fig. 5.**
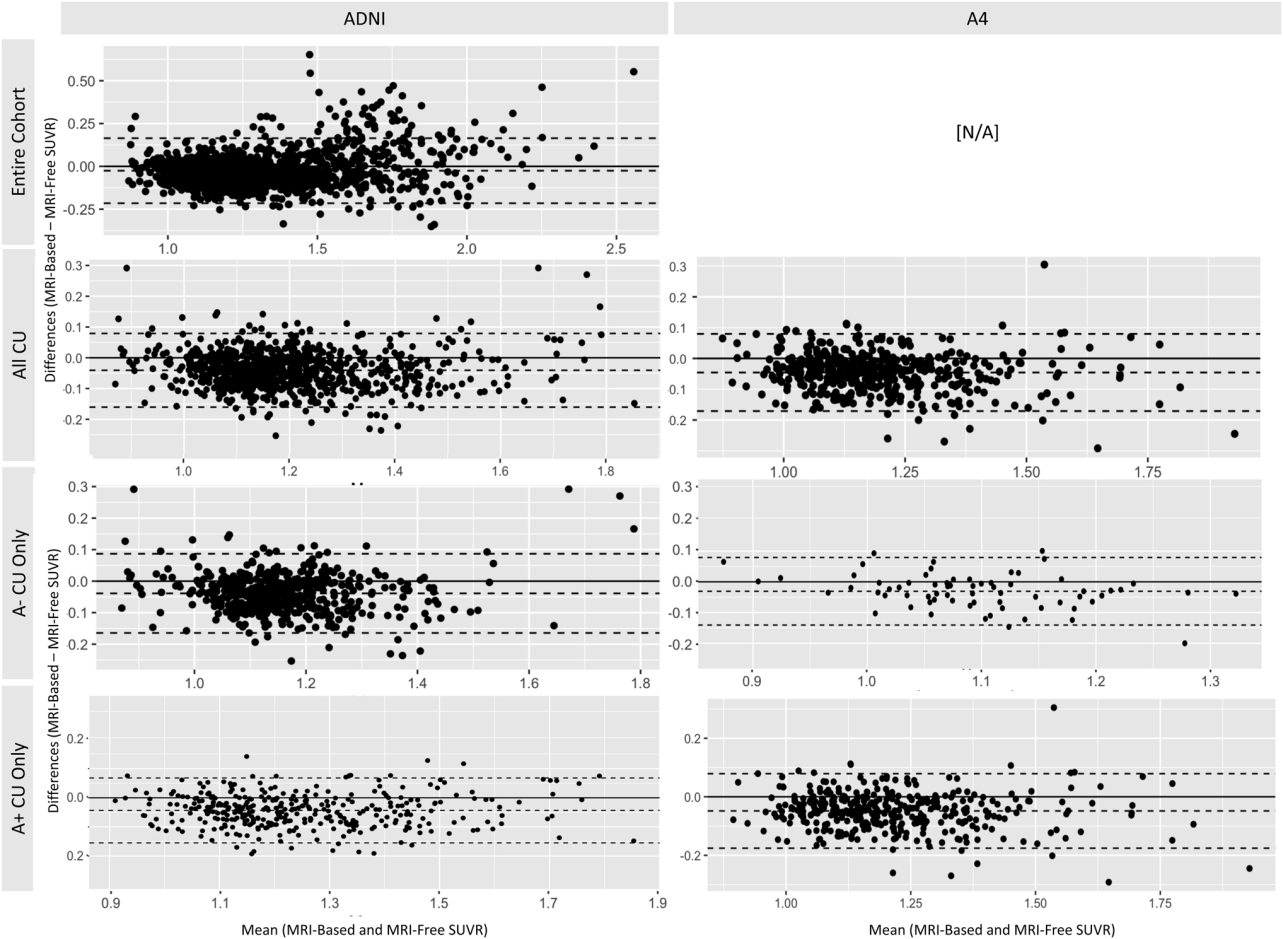
Bland-Altman plots, using entorhinal cortex as an illustrative region of interest, showing differences between the means of MRI-based and MRI-free baseline tau SUVR in each cohort, altogether and CU grouped by amyloid status.

**Fig. 6.**
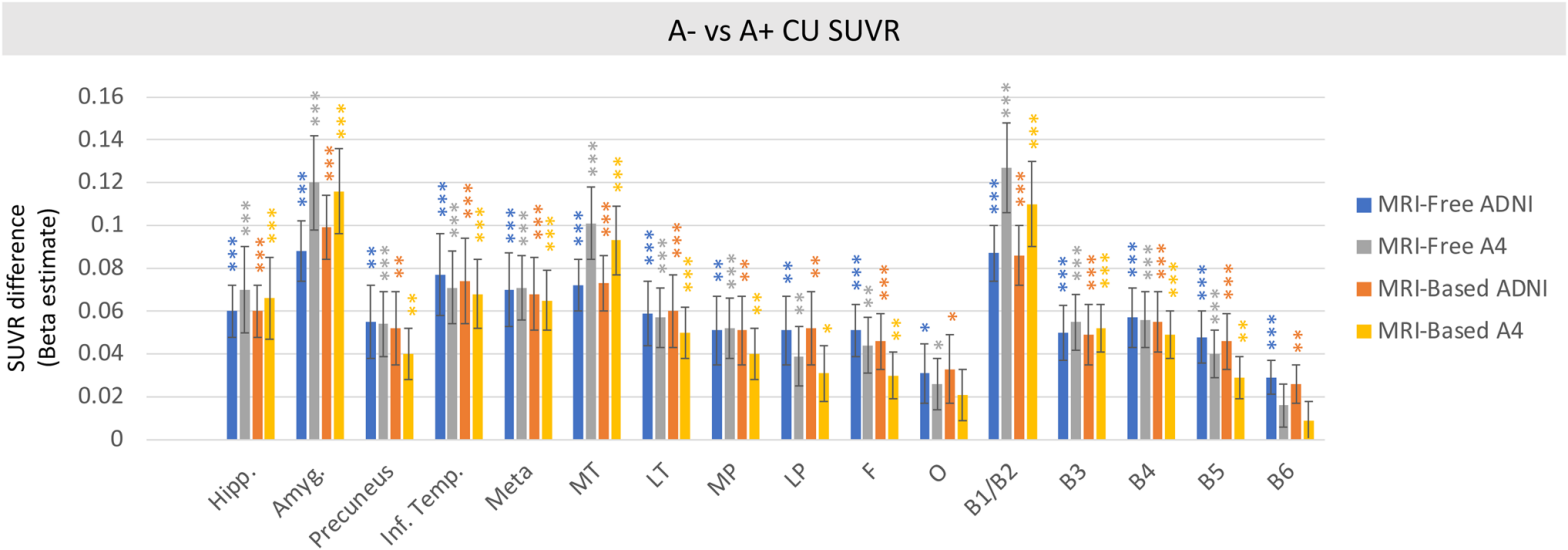
MRI-free and MRI-based cross-sectional differences in beta weights between preclinical groups in regions of early tau accumulation. Stars indicate significant difference between A- CU and A+ CU. (***P < 0.001, **P < 0.01, *P < 0.05). Meta = meta temporal composite region, MT = medial temporal, MP = medial parietal, LT = lateral temporal, LP = lateral parietal, F = frontal, O = occipital.

In nearly all tau-specific ROIs across all cohorts, MRI-free processing was able to detect early significant differences between mean tau PET SUVR in A- CU compared to A+ CU individuals (Fig. 6). As expected, differences between A- CU and A+ CU groups were largest in the earliest regions of pathologic tau accumulation, including medial temporal lobe structures (Supplementary Table 4). Large differences with MRI-free SUVRs were also noted across the clinical spectrum (Supplementary Table 5).

**Fig. 7.**
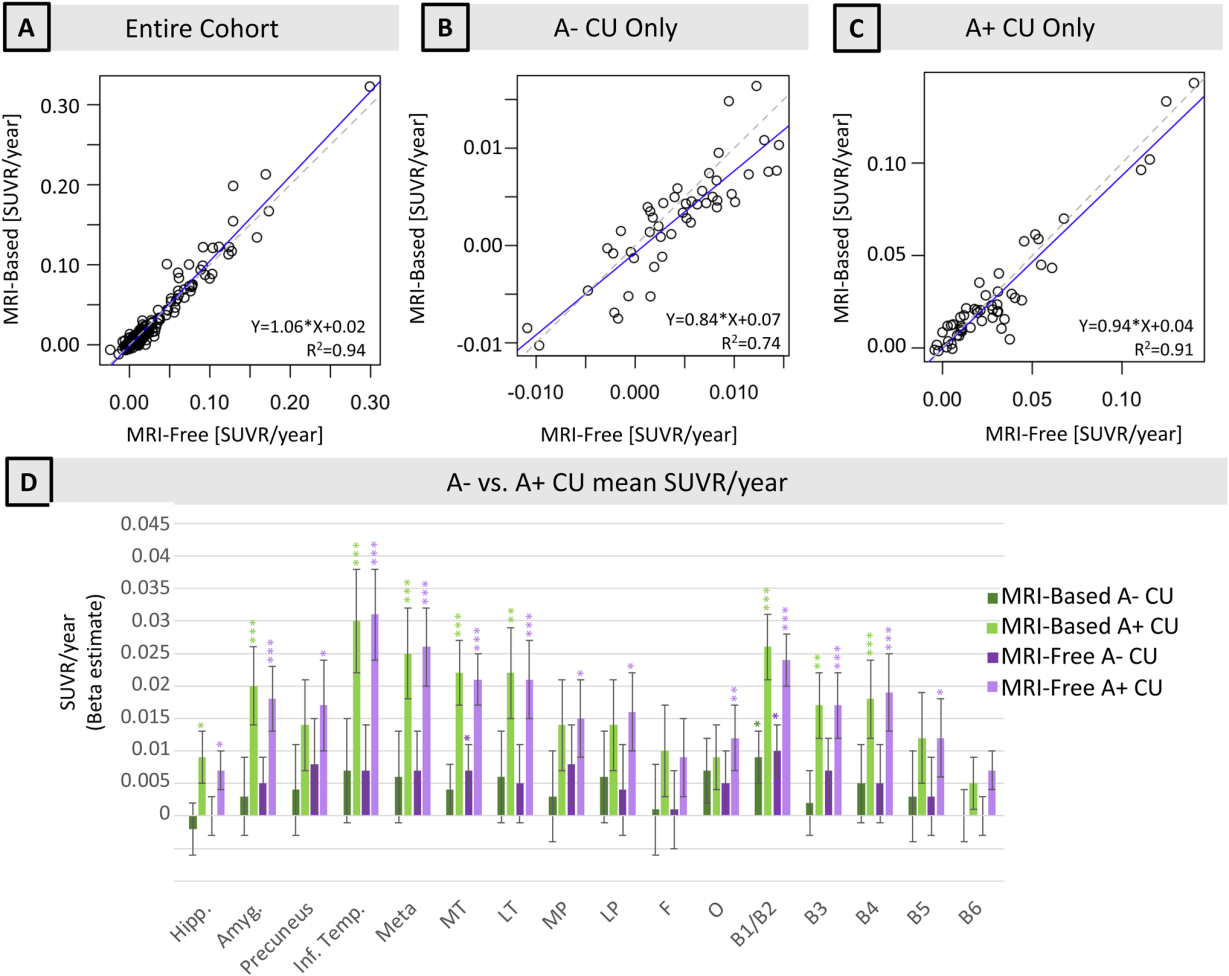
Annual change in SUVR in the inferior temporal cortex of (A) all longitudinal participants, (B) A- CU only, and (C) A+ CU only. (D) Beta weights of annual change in SUVR in all ROIs using both pipelines. Stars indicate significant difference in SUVR/year from no change, and standard error is listed inSupplementary Table 6. Meta = meta temporal composite region, MT = medial temporal, MP = medial parietal, LT = lateral temporal, LP = lateral parietal, F = frontal, O = occipital. In (A–C), blue line is linear regression and dashed line is identity.

Using longitudinal ADNI participants with 3+ tau PET scans, we computed tau PET SUVR/year in each ROI for each diagnostic group (Supplementary Table 6). MRI-free processing produced comparable rates of change compared to MRI-based processing. Pipeline-related differences in SUVR/year were not significant in any region of interest (Supplementary Table 7). MRI-free annual change in SUVR was associated with the MRI-based SUVR/year (0.46 < R^2^< 0.97) across all regions, even when examining the association in A- CU and A+ CU separately (Fig. 7;Supplementary Table 7).

Using MRI-free values, there were significant differences in SUVR/year between A- CU and A+ CU participants in the entorhinal cortex, hippocampus, amygdala, inferior temporal cortex, and composite regions meta temporal and medial temporal, and rates of tau accumulation in A+ CU participants were significantly positive in many regions of early expected tau accumulation (Fig. 7D). A- MCI compared to A+ MCI participants also had more significant differences in tau SUVR/year in regions of both early and later tau accumulation, including Braak ROIs I-IV (P < 0.02 for all) (Supplementary Table 8).

## 4 Discussion

We show that an MRI-free pipeline that leverages multiple templates reflecting different tau PET binding patterns provides regional tau PET values that are consistent to those extracted with MRI-based pipelines with both cross-sectional and longitudinal data. Importantly, MRI-free regional tau PET values show cross-sectional and longitudinal differences among A- and A+ CU individuals, highlighting that simplified processing pipelines show promise for efforts geared towards early detection.

MRI-free pipelines for amyloid PET processing have been developed and demonstrate consistency with MRI-based approaches (Bourgeat et al., 2015;Doré et al., 2019;Edison et al., 2013;Iaccarino et al., 2022;Landau et al., 2023;Pegueroles et al., 2021). Fewer studies have applied these pipelines to tau PET, though recent work has demonstrated promising results (Fleisher et al., 2020;Landau et al., 2023;Ward et al., 2023). Given that tau PET patterns are focal during the initial stages of AD, (Schöll et al., 2016) show heterogenous patterns of uptake in some individuals (Leuzy et al., 2019;Ossenkoppele et al., 2016;Younes et al., 2024;Young et al., 2022), and have various sources of focal off-target binding such as in choroid plexus, basal ganglia, and meninges (Baker et al., 2019;Harrison et al., 2023;Pascoal et al., 2020), it is possible that MRI-free pipelines may be more prone to errors for tau PET, which is often measured in relatively small regions, compared to more globally distributed amyloid PET. In the study byLandau et al. (2023), the correspondence of longitudinal rates of change in tau SUVR between pipelines was lower (R^2^= 0.75 to 0.90) than cross-sectional comparisons, suggesting that the measurement of subtle changes over time may be influenced by processing pipeline. However, MRI-free differences in tau PET among CU individuals had not been evaluated. We, therefore, sought to evaluate MRI-free tau PET pipelines specifically in the context of clinically unimpaired (CU) individuals, where it is expected that tau PET values will have a more restricted range and biologically relevant elevations will be more subtle than what is observed among individuals with clinical impairment (mild cognitive impairment and dementia). Given the restricted range of elevations among CU, we reasoned that the ability to detect differences within this group may be more sensitive to pipeline differences. We evaluated the performance of MRI-free tau PET processing by examining 1) the correspondence between MRI-free SUVRs with MRI-based SUVRs among A- and A+ CU groups and 2) whether cross-sectional and longitudinal differences are detectable between A- CU and A+ CU groups using MRI-free pipelines.

We found that MRI-free SUVRs were highly correlated MRI-based SUVRs, with the entire ADNI dataset but also when restricted to the ADNI CU sample as well as in an independent CU cohort (A4). We found that correspondences between MRI-free and MRI-based values were systematically lower although still high in the independent cohort compared to ADNI, highlighting the importance of evaluating new pipelines in independent datasets. In the independent A4 dataset of CU, ICCs ranged from 0.69–0.93, indicating moderate to excellent correspondence (Koo & Li, 2016). We also found that the multi-template approach systematically resulted in higher correspondence than the generic template in both ADNI and A4. Among all 16 ROIs examined, there was an average ICC of 0.90 for the ADNI CU group and 0.81 for the A4 CU group. The improved performance of the multi-template approach may reflect a more precise alignment into template space, which may be particularly important for CU cohorts that demonstrate focal patterns up uptake. When assessing the correlation between MRI-free and MRI-based pipelines, we found a systematic shift in SUVR magnitude, such that the MRI-free pipeline generated higher values than corresponding MRI-based SUVRs. It is possible that these differences are driven by reference region and/or target regions. Given the high correlation, this shift could presumably be corrected by applying a regression equation to MRI-free SUVRs to combine these values with MRI-based SUVRs within a given dataset. For instance, although some PET studies include MRI as part of their study protocol, it is possible this datatype may be missing for a given visit due to technical errors or scheduling conflicts during the planned MRI acquisition. In this case, an investigator may still choose to include the tau PET data processed with an MRI-free pipeline, applying a regression-based shift to enable this datapoint to be combined with the larger dataset processed with an MRI-Based pipeline

After establishing a high correlation between MRI-free and MRI-based pipelines, we sought to determine whether MRI-free SUVRs can detect significant differences among CU individuals. Overall, we found that both cross-sectional and longitudinal SUVR differences were detectable between A- CU and A+ CU individuals using MRI-free processing across most ROIs and that these group differences were similar to those found with MRI-based processing. The ability to quantify subtly elevated tau PET signal in CU groups is particularly important for early detection efforts. Tau PET studies in CU cohorts consistently show elevated signal in the medial temporal lobe as well as neocortical regions such as inferior temporal cortex and medial parietal cortex in A+ CU compared to A- CU (Betthauser et al., 2020;Lockhart et al., 2017;Sperling et al., 2019;Vemuri et al., 2017). Importantly, focal tau PET signal among CU has been critical in understanding rates of future progression to mild cognitive impairment and/or dementia (Cai et al., 2023;Ossenkoppele, Pichet Binette, et al., 2022), as well as in explaining subtle differences in memory (Lowe et al., 2019;Maass et al., 2019;Sperling et al., 2019). Thus, the ability to accurately measure regional increases in CU individuals using simplified MRI-free processing of tau PET data has important implications for clinical trial design and for the real world setting if disease modifying treatments become available for CU individuals (Updated Appropriate Use Criteria for Amyloid and Tau PET in Alzheimer’s Disease,Rabinovici, et al., 2023).

Given the importance of early tau PET signal and the potential for this modality to be used in a real-world setting, it is important to specifically evaluate the performance of simplified MRI-free pipelines early in disease. Eliminating the need for anatomic imaging to aid in PET registration would enable patients with MRI contraindications to still undergo quantitative PET imaging. Such contraindications as metallic implants or artificial joints, pacemakers, aneurism clips, cochlear implants, and claustrophobia are more common in aging adults. Clinics would also reduce the time and costs associated with MRI scanning if only PET is needed.

## 5 Limitations/Future Work

There are a few limitations to this work. First, a limitation of this work is the lack of a gold standard method for comparing quantitative results from MRI-free processing. We treated MRI-based values as the gold standard since one of the most important reasons for MRI-free processing is clinical use, and to address this we only needed to determine whether MRI-free is as good as MRI-dependent processing. Another limitation is that MRI-free SUVR correspondence with visual reads was not evaluated, which may be a more applicable method of validation in a real-world setting. Third, we have not compared pipeline performance on different acquisition windows. In this study, A4 data have an acquisition window that is 5 minutes later than the ADNI window, and while we would not expect a significant impact on results, this could still be investigated in the future. Although we note a systematic shift in SUVR values computed from the MRI-based and MRI-free pipelines, it is unclear whether these differences are driver by the reference region, target region, and/or reflect differences between extractions done in template versus native space. Lastly, although we established that similar amyloid group effects were present across pipelines, we did not comprehensively evaluate the impact of pipeline on other subtle tau PET effects that have been reported in the literature, such as sex effects (Buckley et al., 2019). We also did not evaluate the impact of pipeline on off target binding and whether contamination of off target binding into target regions varies by pipelines (Flores et al., 2023). Future work focused on such effects may need to establish whether these effects are confounded by pipeline selection.

## 6 Conclusion

Multi-template MRI-free processing of cross-sectional tau PET images yields SUVR values highly comparable to MRI-based values in multiple regions of interest used throughout the literature. This method can capture subtle cross-sectional and longitudinal differences in SUVR accumulation over time in A+ CU individuals. This work highlights that simplified MRI-free processing can be applied to tau PET data to detect subtle changes in preclinical AD.

## Supplementary Material

Supplementary Material

## Data Availability

Code for the MRI-free processing pipeline, along with PET templates, is available upon request.
