# Supplementary material for: MRI-free processing of tau PET images for early detection
Source: Imaging Neurosci (Camb). 2024 Nov 13;2:imag-2-00369. doi: 10.1162/imag_a_00369 (PMC12315776; doi:10.1162/imag_a_00369)
Supplement: Supplementary Material [file imag_a_00369-supp.pdf]

SUPPLEMENT: MRI-Free Processing and Analysis of Tau PET images

Abbreviations

CL: Centiloid

FBB: Florbetaben

FBP: Florbetapir

FTP: Flortaucipir

PIB: Pittsburgh Compound-B

SUVR: Standardized Update Value Ratio

Supplemental Information

S. Methods 1: CL overview.

S. Figure 1: FBP CL calculation.

S. Figure 2. FBB CL calculation.

S. Figure 3. PIB CL calculation.

S. Table 1: CL equations.

S. Figure 4: CL conversion flowchart.

S. Figure 5: CL distributions for each cohort.

S. Methods 2: MRI-Free Multi-tracer Template Pipeline Optimization.

S. Figure 6: Comparison of XC15 and XC30.

S. Table 2: ROI definitions.

S. Figure 7: R^2 correlations between MRI-based and MRI-free SUVRs.

S. Figure 8: Scatterplots between MRI-Free and MRI-Based SUVRs.

S. Table 3: Mean SUVR differences between MRI-Free and MRI-Based pipeline SUVRs.

S. Table 4: Effect sizes (Cohen's D) in SUVR differences between A- CU and A+ CU groups, by pipeline (MRI-Free and MRI-Based).

S. Table 5: Cross-sectional differences between diagnostic groups.

S. Table 6: Longitudinal annual change in tau PET SUVR within each diagnostic group.

S. Table 7: Impact of pipeline on annual change.

S. Table 8: Longitudinal annual change differences in tau PET SUVR between diagnostic groups.

S. Methods 1: CL overview.

We derived tracer-specific SUVR-to-CL equations for our MRI-free pipeline using the level 2 GAAIN CL analysis method (Klunk et al., 2015). Florbetapir (FBP): we downloaded and processed the Navitsky et al. (Navitsky et al., 2018) paired 50-70 minute Pittsburgh Compound-B (PIB) dataset and 50-60 minute FBP dataset (N=46; 9 older CU, 13 younger CU, 7 MCI, and 18 AD). Florbetaben (FBB): we downloaded and processed the Rowe et al. (Rowe et al., 2017) paired 50-70 minute PIB and 90-110 minute FBB dataset [N=35; 6 older CU, 10 younger CU, 9 MCI, 8 AD, and 2 frontotemporal dementia]. In these “paired” datasets, each individual is scanned with both ligands, which enables direct conversion from the F18 ligand to the corresponding PIB value. FBP (Navitsky et al., 2018) and FBB scans (Rowe et al., 2017) and were processed locally using the MRI-Free pipeline described in the manuscript (Landau et al., 2023). A linear regression was performed between the resulting MRI-Free FBB (or FBP) SUVRs and their corresponding “standardized” PiB SUVRs. Standardized PiB SUVRs were not calculated by our lab but instead are downloaded from https://www.gaain.org/centiloid-project. Equations were derived for the global SUVRs normalized to the whole cerebellum SUVR, enabling conversion from our FBB and FBP MRI-Free SUVRs to "PiB calculated" SUVRs. Subsequently, we scaled the "PiB calculated" SUVRs into CLs using the mean of 34 young CU (“YC-0”; mean=1.01) and 45 AD (“AD-100”; mean=2.08) (Klunk et al., 2015) (**S. Figures 1-2**).

PIB: A similar process was done for converting MRI-Free PIB processed data to centiloids. This was completed using the 79 PIB scans used in Klunk et al. (Klunk et al., 2015) [34 younger CU and 45 AD]. These 79 scans were downloaded and processed with our MRI-Free pipeline. Similar to the steps described for FBP and FBB, a linear regression was performed between the resulting

MRI-Free PIB SUVRs and their corresponding “standardized” PIB SUVRs. These transformed PIB SUVRs were then scaled by the mean of the YC-0 and AD-100 groups (**S. Figure 3**).

For each tracer, we derived the positivity threshold by converting the SUVR positivity threshold provided by the SCAN Core at UC Berkeley that is currently used for SCAN compliant PET scans collected across NACC Alzheimer’s Disease Research Centers (ADRCs) (Murphy et al., 2022). Given slight differences in processing between UC Berkeley and Stanford, we converted these values following steps described in **S. Figure 4**, and the SUVR to CL conversions are listed in **S. Table 1**.

S. Figure 1: FBP CL calculation.

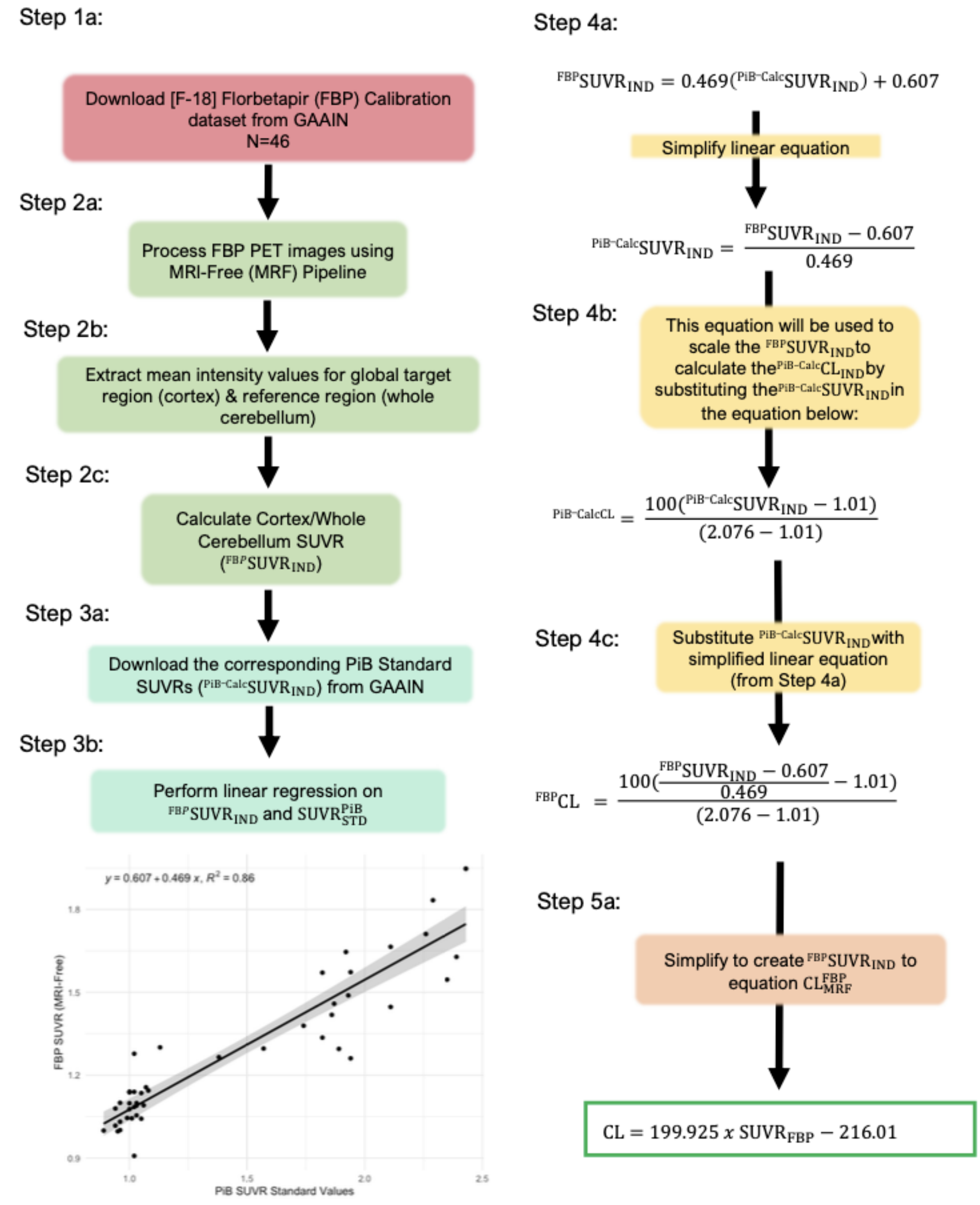

S. Figure 2. FBB CL calculation.

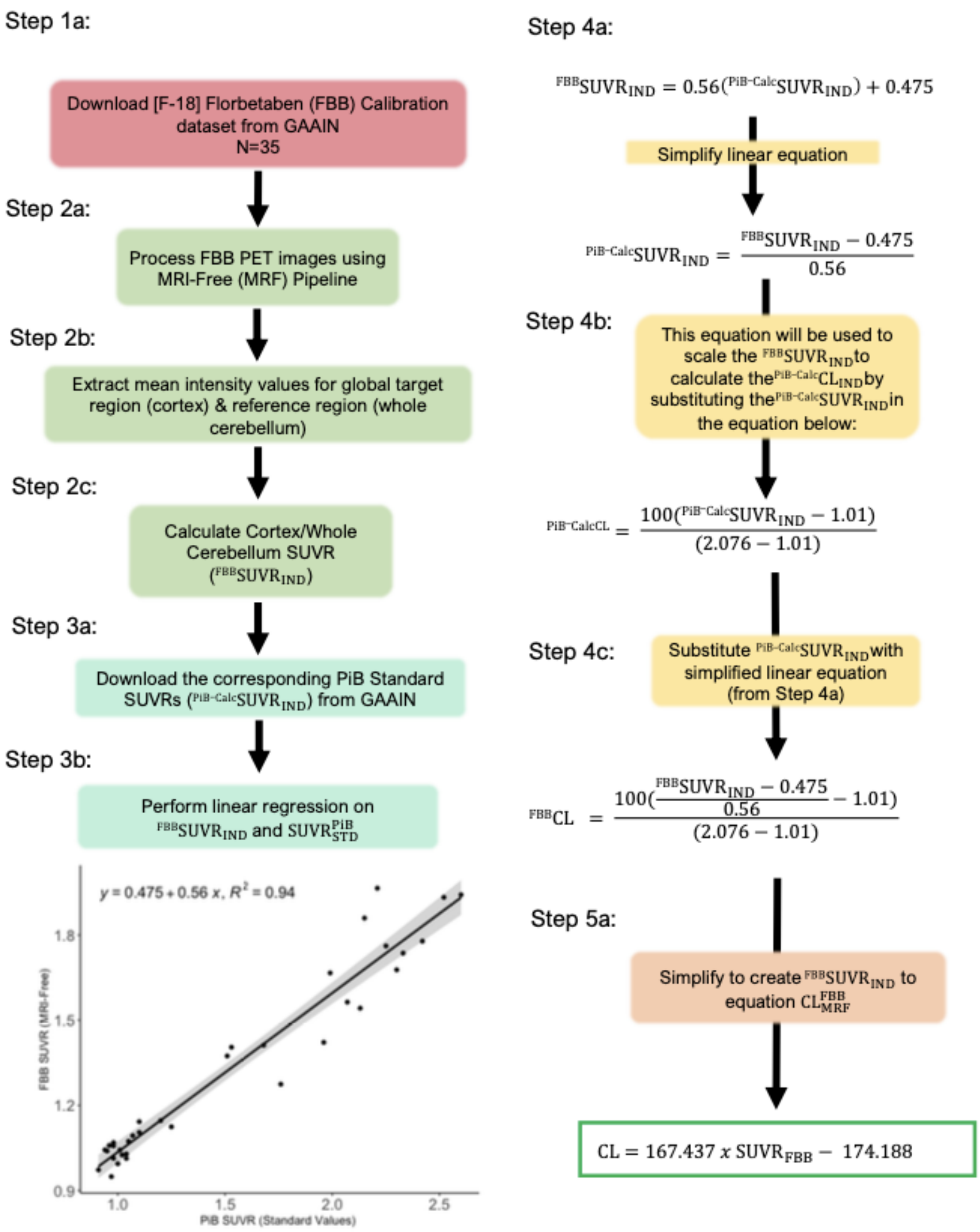

S. Figure 3. PIB CL calculation.

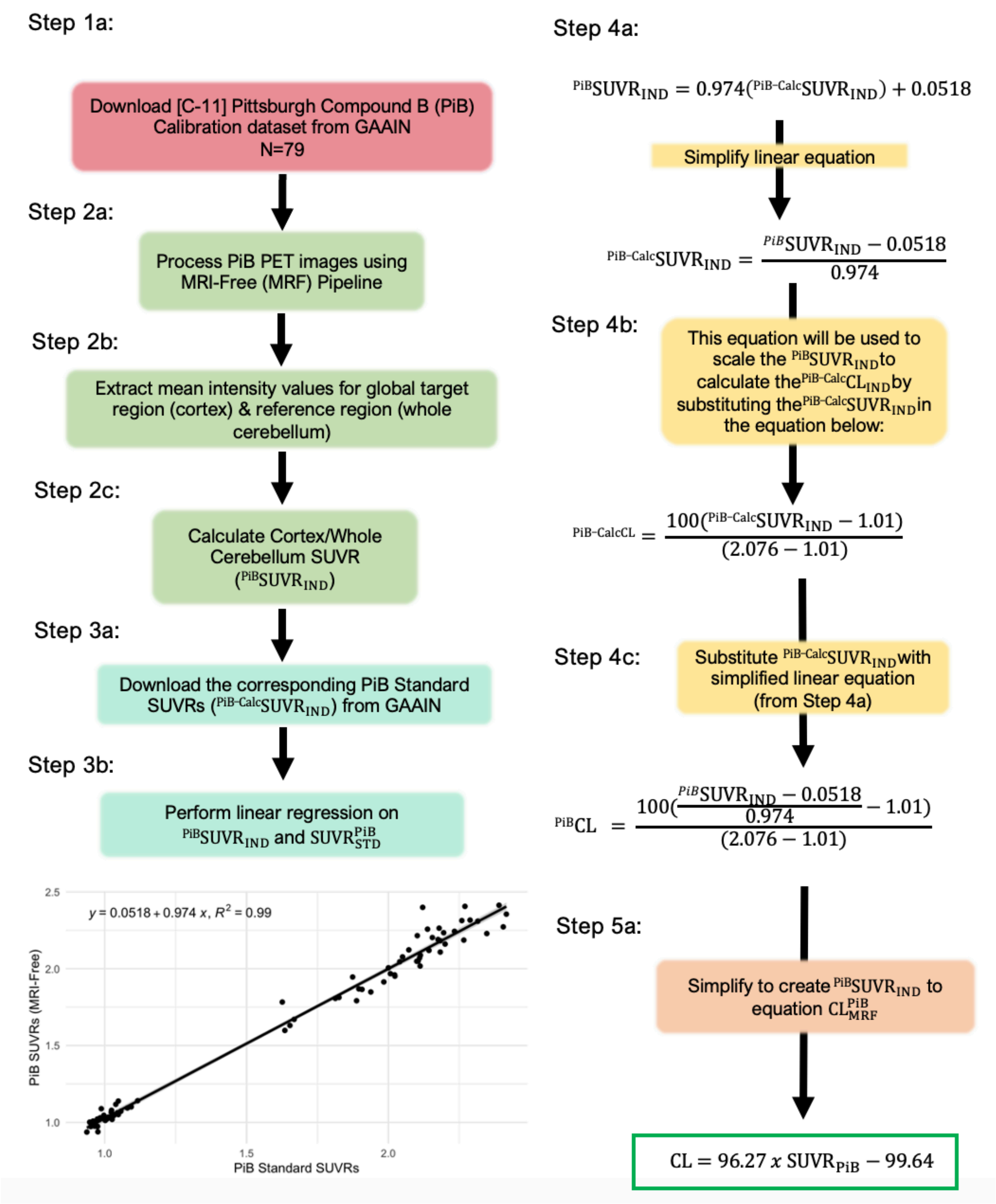

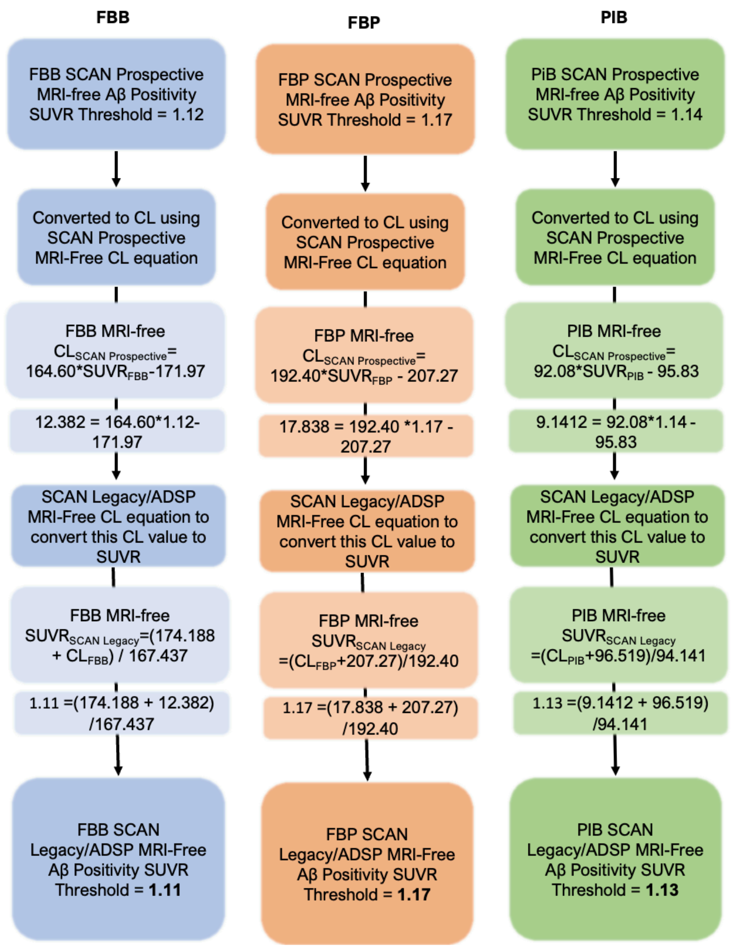

S. Figure 4: Calculation of Stanford cut offs based on slight differences with Berkeley MRI-Free CL equations.

S. Table 1: CL equations.

Tracer	MRI-Free SUVR Cutoff	MRI-free SUVR → MRI-free CL	MRI-Free CL Cutoff
FBP	1.17	(FBP MRI-free CL) = 199.925 (MRI-free FBP SUVR)-216.01	18
FBB	1.11	(FBB MRI-free CL) = 167.437 (MRI-free FBB SUVR) - 174.188	12
PIB	1.13	(PiB MRI-free CL) = 96.27(MRI-free PiB SUVR) – 99.64	9

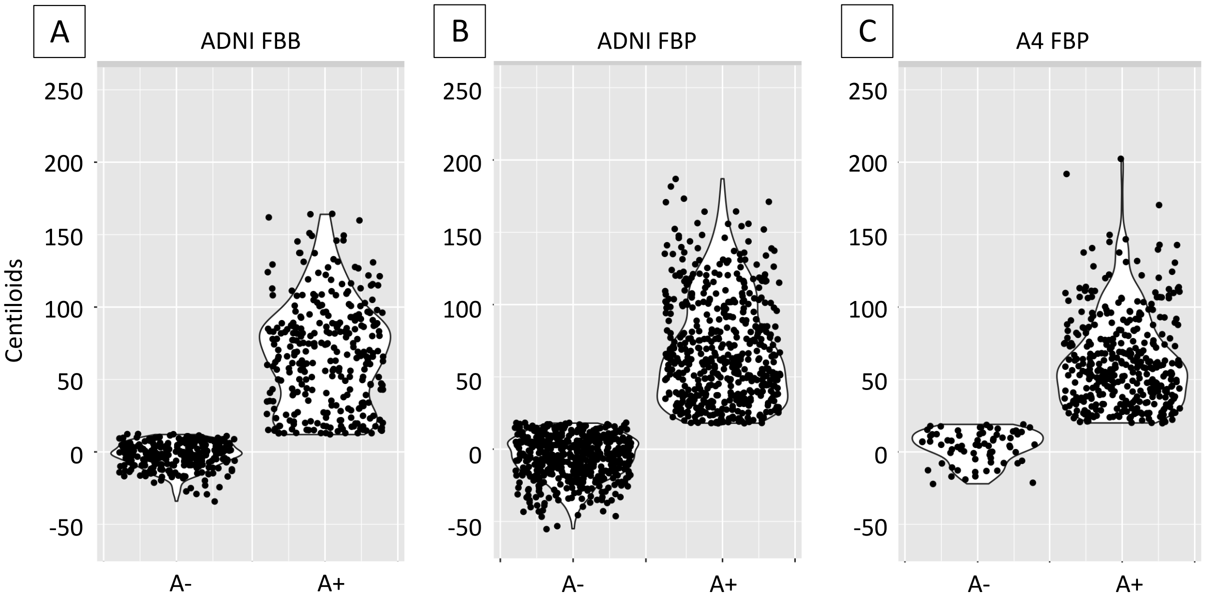

S. Figure 5: CL distributions for each cohort. ADNI PiB is not depicted given that there were only 2 participants with PiB data.

S. Methods 2: MRI-Free Multi-Template Pipeline Optimization.

We explored whether the performance of the multi-template approach used in spatial normalization differed based on the number of scans used to create these templates. We created FTP templates using 15 subjects per template (XC15) and another set of templates using 30 subjects per template (XC30). All subjects in each of the four diagnostic groups were randomly selected from ADNI. All ADNI data were processed with the MRI-Free multi-template pipeline twice, once with the XC15 templates and then again with the XC30 templates. We then examined the association between XC15 and XC30 SUVRs in six early tau-specific ROIs (bilateral entorhinal cortex, hippocampus, amygdala, precuneus, inferior temporal cortex, and meta temporal region). Correlation between these approaches had an $R^2 > 0.99$, slopes near 1, and intercepts near 0 (**S.**

Figure 6). Given this high similarity, the XC15 was used as the primary approach in the manuscript.

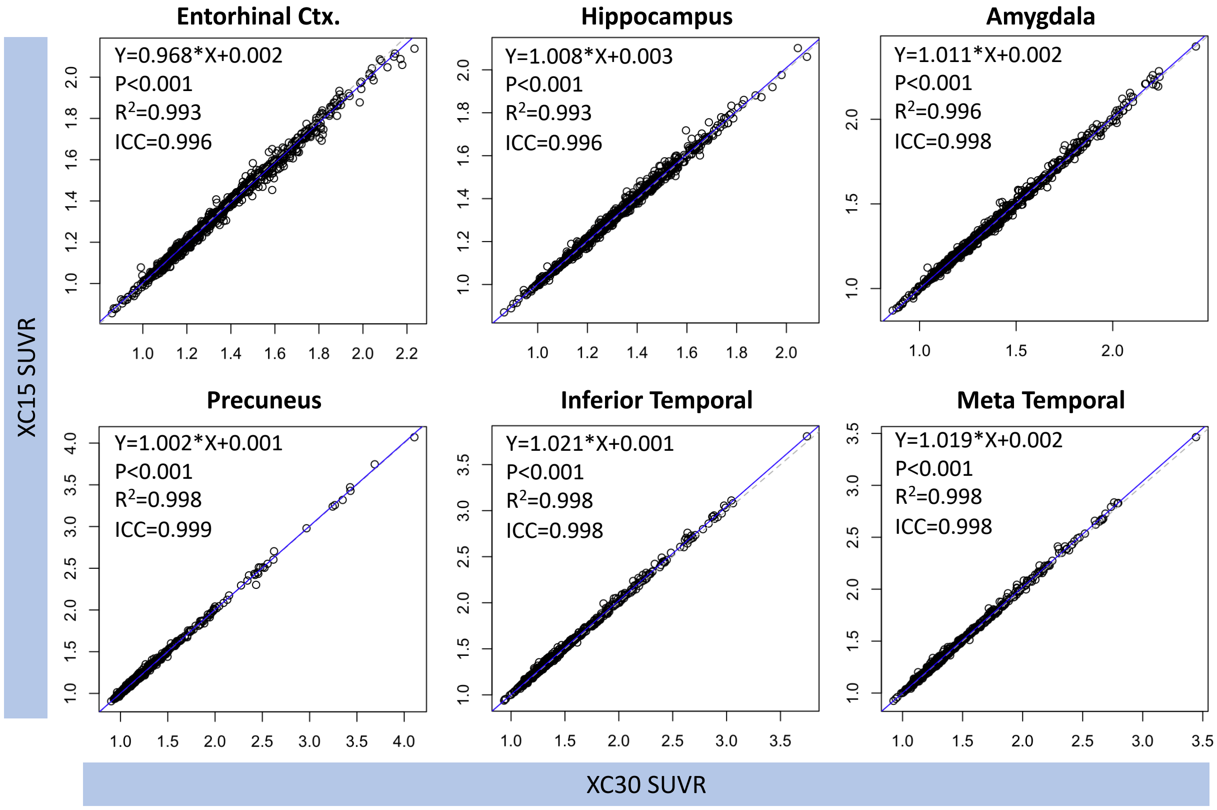

S. Figure 6: Comparison of XC15 and XC30.

Default parameters to linearly coregister source PET image to T1 MRI in MNI space:

```
cost_fun = 'nmi';
sep = [4 2];
tol = [0.02 0.02 0.02 0.001 0.001 0.001 0.01 0.01 0.01 0.001 0.001 0.001];
fwhm = [7 7];
interp = 0;
wrap = [0 0 0];
mask = 0;
```

Default parameters to warp individual PET (source image) to PET templates using SPM's oldnorm toolbox:

```
smosrc = 8;
smoref = 0;
regtype = 'mni';
```

```
cutoff = 25;
nits = 16;
reg = 1;
preserve = 0;
bb = [-90 -126   -72
          91    91  109];
vox = [1 1 1];
interp = 0;
wrap = [0 0 0];
```

S. Table 2: ROI definitions.

Scheme	ROIs (Bilateral)
Early Tau	Hippocampus
	Amygdala
	Precuneus
	Inferior temporal cortex
	Meta-temporal: entorhinal, fusiform, middle temporal, amygdala, and inferior temporal cortices
Cortical	Medial temporal: entorhinal and parahippocampal cortex
	Lateral temporal: superior, middle, and inferior temporal cortex
	Medial parietal: posterior cingulate cortex and precuneus
	Lateral parietal: inferior and superior parietal and supramarginal
	Frontal: superior frontal, rostral and caudal middle frontal, and lateral and medial orbitofrontal cortex
	Occipital: pericalcarine, cuneus, and lateral occipital cortex
Braak-Based	Braak I and II (B I/II): entorhinal cortex
	Braak III (B III): amygdala, parahippocampal gyrus, fusiform gyrus and lingual gyrus
	Braak IV (B IV): insula, inferior temporal, lateral temporal, posterior cingulate and inferior parietal cortex
	Braak V (B V): orbitofrontal, superior temporal, inferior frontal, cuneus, anterior cingulate, supramarginal gyrus, lateral occipital, precuneus, superior parietal, superior frontal and rostromedial frontal cortex
	Braak VI (B VI): paracentral, postcentral, precentral and pericalcarine

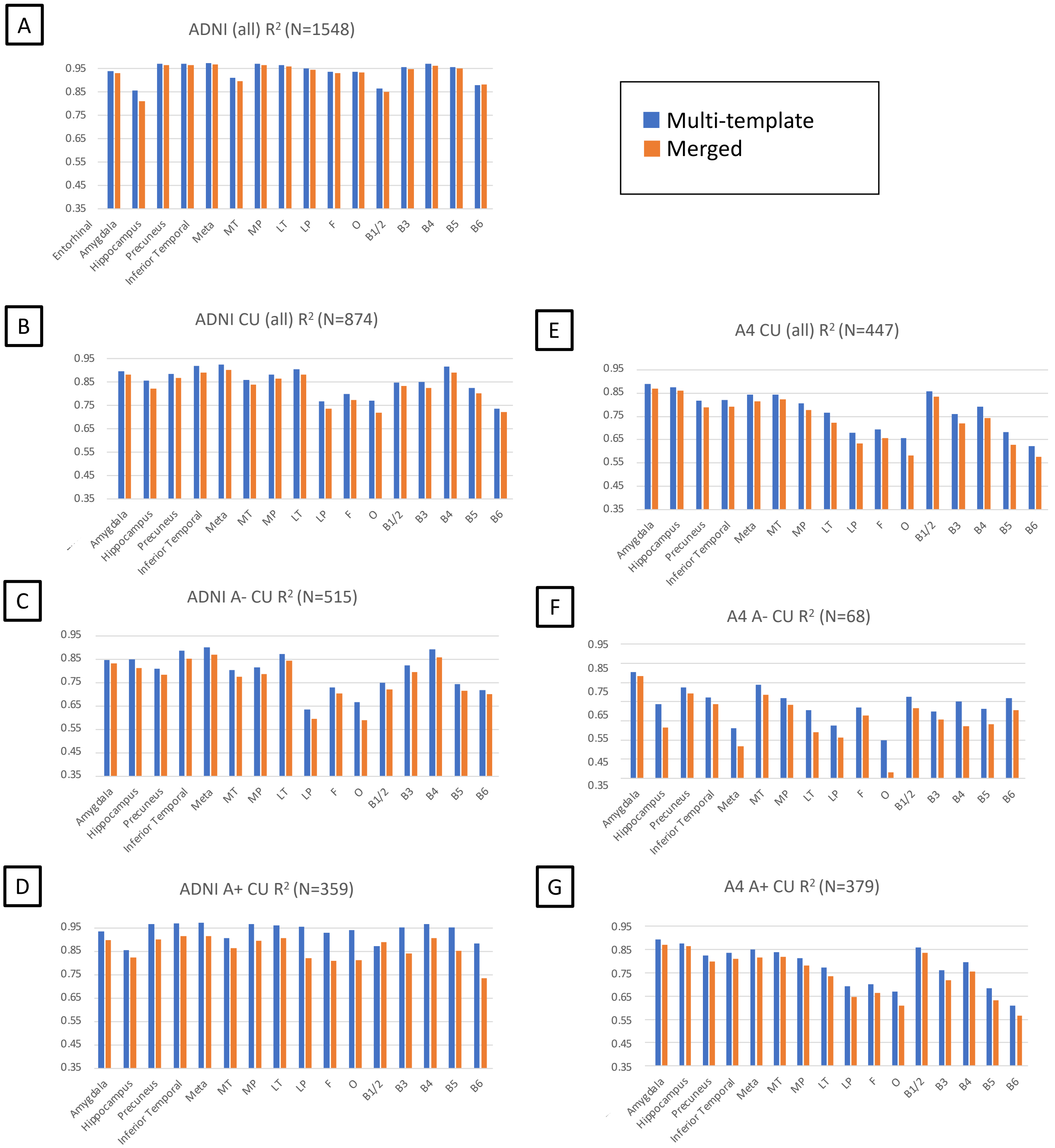

S. Figure 7: Association between MRI-based and MRI-free SUVRs in tau-relevant ROIs for ADNI (A-D) and A4 (E-F).

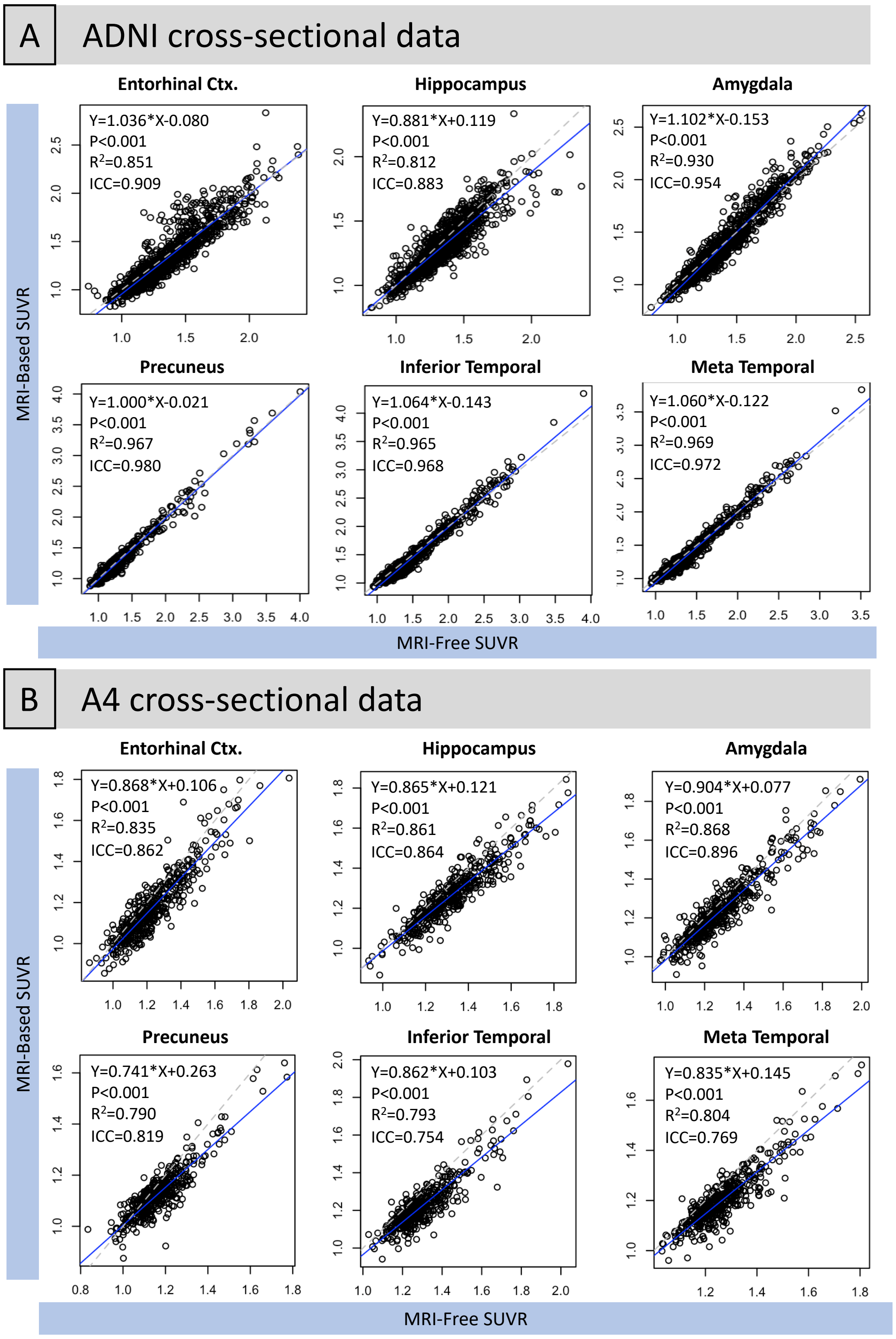

S. Figure 8: Association between merged-template MRI-Free and MRI-Based SUVR in regions of early tau accumulation in the entire cohort of (A) ADNI and (B) A4 data.

S. Table 3: Mean SUVR differences between MRI-Free and MRI-Based pipeline SUVRs. Mean difference and standard error for pairwise differences across pipelines are displayed, colored by the p-value result of each T-test, as well as the pipeline term from a linear regression predicting SUVRs. The MRI-Free pipeline resulted in slightly higher values than the MRI-Based pipelines.

			Early Medial Temporal Lobe ROIs					Composite Cortical ROIs						Braak Stage-Based ROIs				
		**Group**	**Hipp.**	**Amyg.**	**Precuneus**	**Inf. Temp.**	**Meta**	**MT**	**LT**	**MP**	**LP**	**F**	**O**	**B1/B2**	**B3**	**B4**	**B5**	**B6**
T-Test Mean Differences	ADNI [^{18}F]FTP	A- CU	0.043 (0.002)	0.024 (0.002)	0.012 (0.002)	0.045 (0.002)	0.036 (0.002)	0.035 (0.002)	0.018 (0.002)	0.012 (0.002)	0.021 (0.003)	0.013 (0.002)	0.027 (0.002)	0.041 (0.003)	0.032 (0.002)	0.026 (0.002)	0.014 (0.002)	0.025 (0.002)
		A+ CU	0.045 (0.004)	0.018 (0.003)	0.017 (0.002)	0.052 (0.003)	0.042 (0.002)	0.036 (0.003)	0.020 (0.002)	0.015 (0.002)	0.022 (0.003)	0.019 (0.003)	0.027 (0.003)	0.044 (0.003)	0.035 (0.002)	0.032 (0.002)	0.018 (0.002)	0.029 (0.002)
		A- MCI	0.033 (0.005)	0.017 (0.005)	0.014 (0.003)	0.045 (0.004)	0.035 (0.003)	0.026 (0.005)	0.013 (0.004)	0.014 (0.003)	0.014 (0.004)	0.010 (0.004)	0.021 (0.004)	0.028 (0.006)	0.034 (0.003)	0.023 (0.003)	0.011 (0.003)	0.021 (0.004)
		A+ MCI	0.010 (0.005)	-0.011 (0.005)	0.025 (0.004)	0.066 (0.004)	0.048 (0.003)	0.015 (0.005)	0.029 (0.004)	0.025 (0.003)	0.021 (0.004)	0.017 (0.004)	0.033 (0.005)	0.019 (0.007)	0.034 (0.004)	0.048 (0.003)	0.020 (0.003)	0.031 (0.003)
		A+ AD	-0.032 (0.007)	-0.030 (0.007)	0.025 (0.007)	0.060 (0.007)	0.044 (0.006)	-0.004 (0.008)	0.016 (0.007)	0.025 (0.006)	0.006 (0.007)	-0.007 (0.005)	0.022 (0.008)	-0.027 (0.012)	0.033 (0.005)	0.052 (0.005)	0.007 (0.005)	0.021 (0.004)
	A4 [^{18}F]FTP	A- CU	0.033 (0.006)	0.024 (0.006)	0.020 (0.005)	0.052 (0.007)	0.043 (0.005)	0.030 (0.005)	0.038 (0.006)	0.018 (0.005)	0.031 (0.007)	0.024 (0.007)	0.046 (0.007)	0.031 (0.007)	0.036 (0.005)	0.038 (0.005)	0.028 (0.006)	0.031 (0.005)
		A+ CU	0.037 (0.003)	0.028 (0.003)	0.034 (0.003)	0.054 (0.003)	0.049 (0.002)	0.038 (0.003)	0.045 (0.003)	0.030 (0.002)	0.039 (0.003)	0.038 (0.003)	0.051 (0.003)	0.048 (0.003)	0.040 (0.003)	0.045 (0.002)	0.039 (0.003)	0.039 (0.002)
Linear Model	interactions	**ADNI**	0.029 (0.006)	0.009 (0.007)	**0.016 (0.009)**	0.049 (0.010)	0.038 (0.009)	0.024 (0.006)	0.017 (0.008)	**0.015 (0.008)**	**0.016 (0.009)**	**0.012 (0.006)**	0.027 (0.008)	0.024 (0.007)	0.033 (0.007)	0.032 (0.007)	0.014 (0.007)	0.025 (0.004)
		**A4**	0.037 (0.010)	0.027 (0.011)	0.032 (0.007)	0.054 (0.009)	0.048 (0.007)	0.037 (0.009)	0.044 (0.007)	0.028 (0.007)	0.037 (0.007)	0.036 (0.006)	0.050 (0.006)	0.046 (0.011)	0.039 (0.006)	0.044 (0.006)	0.037 (0.005)	0.038 (0.005)

P<0.001 P<0.01 P<0.05

S. Table 3A: Raw versus FDR-corrected P-Values from S. Table 3 illustrating high consistency between pipelines.

			Braak Stage-Based ROIs				
		**Group**	**B1/B2**	**B3**	**B4**	**B5**	**B6**
T-Test Mean Differences	ADNI [^{18}F]FTP	A- CU	< 2.2E-16	< 2.2E-16	< 2.2E-16	< 2.2E-16	< 2.2E-16
		A+ CU	< 2.2E-16	< 2.2E-16	< 2.2E-16	< 2.2E-16	< 2.2E-16
		A- MCI	2.40E-05	< 2.2E-16	6.61E-12	0.001423	6.41E-09
		A+ MCI	0.005881	< 2.2E-16	< 2.2E-16	1.96E-10	< 2.2E-16
		A+ AD	0.03227	6.98E-09	< 2.2E-16	0.1502	4.16E-06
	A4 [^{18}F]FTP	A- CU	2.71E-05	2.23E-09	1.98E-10	7.42E-06	1.43E-07
		A+ CU	< 2.2E-16	< 2.2E-16	< 2.2E-16	< 2.2E-16	< 2.2E-16

FDR-Corrected P-values

			Braak Stage-Based ROIs				
		**Group**	**B1/B2**	**B3**	**B4**	**B5**	**B6**
T-Test Mean Differences	ADNI [^{18}F]FTP	A- CU	1.10E-15	1.10E-15	1.10E-15	1.10E-15	1.10E-15
		A+ CU	1.10E-15	1.10E-15	1.10E-15	1.10E-15	1.10E-15
		A- MCI	3.00E-05	1.10E-15	1.65E-11	1.42E-03	1.07E-08
		A+ MCI	5.88E-03	1.10E-15	1.10E-15	2.45E-10	1.10E-15
		A+ AD	4.03E-02	1.75E-08	1.10E-15	1.50E-01	6.93E-06
	A4 [^{18}F]FTP	A- CU	2.71E-05	5.58E-09	9.92E-10	9.28E-06	2.39E-07
		A+ CU	1.10E-15	1.10E-15	1.10E-15	1.10E-15	1.10E-15

S. Table 4: Effect sizes (Cohen's D) in SUVR differences between A- CU and A+ CU groups, by pipeline (MRI-Free and MRI-Based).

		Early Medial Temporal Lobe ROIs					Composite Cortical ROIs						Braak Stage-Based ROIs				
	**Cohort**	**Hipp.**	**Amyg.**	**Precuneus**	**Inf. Temp.**	**Meta**	**MT**	**LT**	**MP**	**LP**	**F**	**O**	**B1/B2**	**B3**	**B4**	**B5**	**B6**
**MRI-Free**	ADNI	0.379	0.621	0.485	0.481	0.510	0.578	0.473	0.463	0.460	0.523	0.317	0.585	0.465	0.480	0.518	0.364
	A4	0.467	0.734	0.484	0.541	0.616	0.770	0.547	0.498	0.376	0.430	0.281	0.784	0.571	0.582	0.463	0.220
**MRI-Based**	ADNI	0.432	0.675	0.490	0.473	0.508	0.582	0.468	0.480	0.481	0.495	0.343	0.573	0.497	0.500	0.500	0.329
	A4	0.459	0.742	0.423	0.555	0.622	0.749	0.540	0.445	0.312	0.359	0.225	0.707	0.630	0.585	0.386	0.132

S. Table 5: Cross-sectional differences between diagnostic groups.

			Early Medial Temporal Lobe ROIs					Composite Cortical ROIs						Braak Stage-Based ROIs				
		**Group**	**Hipp.**	**Amyg.**	**Precuneus**	**Inf. Temp.**	**Meta**	**MT**	**LT**	**MP**	**LP**	**F**	**O**	**B1/B2**	**B3**	**B4**	**B5**	**B6**
**MRI-Free**	**ADNI $[^{18}F]$FTP**	A- CU vs A+ CU	0.060 (0.012)	0.088 (0.014)	0.055 (0.017)	0.077 (0.019)	0.070 (0.017)	0.072 (0.012)	0.059 (0.015)	0.051 (0.016)	0.051 (0.016)	0.051 (0.012)	0.031 (0.014)	0.087 (0.013)	0.050 (0.0128)	0.057 (0.0141)	0.048 (0.0123)	0.029 (0.0079)
		A+ CU vs A+ MCI	0.079 (0.014)	0.227 (0.016)	0.129 (0.020)	0.235 (0.023)	0.211 (0.020)	0.189 (0.014)	0.166 (0.018)	0.125 (0.019)	0.120 (0.019)	0.065 (0.014)	0.117 (0.016)	0.223 (0.016)	0.166 (0.0153)	0.158 (0.0168)	0.092 (0.0147)	0.034 (0.0095)
		A+ MCI vs A- MCI	0.150 (0.016)	0.298 (0.018)	0.167 (0.023)	0.289 (0.026)	0.262 (0.022)	0.251 (0.016)	0.216 (0.021)	0.163 (0.022)	0.165 (0.021)	0.119 (0.016)	0.138 (0.018)	0.299 (0.018)	0.197 (0.0173)	0.205 (0.0190)	0.138 (0.0167)	0.064 (0.0107)
		A+ MCI vs A+ AD	-0.014 (0.017)	0.108 (0.019)	0.263 (0.024)	0.311 (0.028)	0.259 (0.024)	0.110 (0.017)	0.232 (0.022)	0.241 (0.023)	0.253 (0.022)	0.140 (0.017)	0.196 (0.020)	0.091 (0.019)	0.187 (0.0184)	0.208 (0.0202)	0.185 ().0177)	0.086 (0.0114)
	**A4 $[^{18}F]$FTP**	A- CU vs A+ CU	0.070 (0.020)	0.120 (0.022)	0.054 (0.015)	0.071 (0.017)	0.071 (0.015)	0.101 (0.017)	0.057 (0.014)	0.052 (0.014)	0.039 (0.014)	0.044 (0.013)	0.026 (0.012)	0.127 (0.021)	0.055 (0.013)	0.056 (0.013)	0.040 (0.011)	**0.016 (0.010)**
**MRI-Based**	**ADNI $[^{18}F]$FTP**	A- CU vs A+ CU	0.060 (0.012)	0.099 (0.015)	0.052 (0.017)	0.074 (0.020)	0.068 (0.017)	0.073 (0.013)	0.060 (0.017)	0.051 (0.016)	0.052 (0.017)	0.046 (0.013)	0.033 (0.016)	0.086 (0.014)	0.049 (0.014)	0.055 (0.014)	0.046 (0.013)	0.026 (0.009)
		A+ CU vs A+ MCI	0.110 (0.014)	0.252 (0.018)	0.127 (0.021)	0.231 (0.024)	0.212 (0.021)	0.213 (0.015)	0.164 (0.020)	0.122 (0.019)	0.128 (0.021)	0.068 (0.015)	0.116 (0.019)	0.253 (0.017)	0.170 (0.016)	0.147 (0.017)	0.094 (0.016)	0.035 (0.011)
		A+ MCI vs A- MCI	0.179 (0.016)	0.328 (0.021)	0.169 (0.024)	0.279 (0.028)	0.261 (0.024)	0.269 (0.018)	0.210 (0.023)	0.164 (0.022)	0.169 (0.024)	0.118 (0.017)	0.138 (0.021)	0.313 (0.020)	0.206 (0.019)	0.191 (0.019)	0.137 (0.018)	0.061 (0.012)
		A+ MCI vs A+ AD	0.025 (0.017)	0.123 (0.021)	0.259 (0.025)	0.305 (0.029)	0.253 (0.025)	0.119 (0.018)	0.239 (0.024)	0.235 (0.023)	0.270 (0.025)	0.162 (0.018)	0.204 (0.022)	0.125 (0.021)	0.182 (0.019)	0.197 (0.020)	0.197 (0.019)	0.094 (0.013)
	**A4 $[^{18}F]$FTP**	A- CU vs A+ CU	0.066 (0.019)	0.116 (0.020)	0.040 (0.012)	0.068 (0.016)	0.065 (0.014)	0.093 (0.016)	0.050 (0.012)	0.040 (0.012)	0.031 (0.013)	0.030 (0.011)	**0.021 (0.012)**	0.110 (0.020)	0.052 (0.011)	0.049 (0.011)	0.029 (0.010)	0.009 (0.009)

P<0.001 P<0.01 P<0.05

S. Table 6: Longitudinal annual change in tau PET SUVR within each diagnostic group. Significant effects reflecting change greater than zero.

		Early Medial Temporal Lobe ROIs					Composite Cortical ROIs						Braak Stage-Based ROIs				
	**Group**	**Hipp.**	**Amyg.**	**Precuneus**	**Inf. Temp.**	**Meta**	**MT**	**LT**	**MP**	**LP**	**F**	**O**	**B1/B2**	**B3**	**B4**	**B5**	**B6**
**MRI-Based**	A- CU (N=43)	-0.002 (0.004)	0.003 (0.006)	0.004 (0.007)	0.007 (0.008)	0.006 (0.007)	0.004 (0.004)	0.006 (0.007)	0.003 (0.007)	0.006 (0.007)	0.001 (0.007)	0.007 (0.005)	0.009 (0.004)	0.002 (0.005)	0.005 (0.006)	0.003 (0.007)	0.000 (0.004)
	A+ CU (N=45)	0.009 (0.004)	0.020 (0.006)	**0.014 (0.007)**	0.030 (0.008)	0.025 (0.007)	0.022 (0.005)	0.022 (0.007)	**0.014 (0.007)**	**0.014 (0.007)**	0.010 (0.007)	**0.009 (0.005)**	0.026 (0.005)	0.017 (0.005)	0.018 (0.006)	**0.012 (0.007)**	0.005 (0.004)
	A- MCI (N=23)	-0.006 (0.006)	0.000 (0.009)	0.006 (0.011)	0.011 (0.011)	0.008 (0.010)	0.001 (0.007)	0.006 (0.010)	0.005 (0.011)	0.008 (0.011)	0.006 (0.010)	0.008 (0.008)	0.004 (0.007)	0.004 (0.008)	0.004 (0.008)	0.006 (0.010)	-0.001 (0.006)
	A+ MCI (N=40)	-0.003 (0.005)	**0.013 (0.007)**	0.027 (0.008)	0.056 (0.009)	0.046 (0.008)	0.024 (0.006)	0.039 (0.008)	0.025 (0.008)	0.030 (0.009)	**0.015 (0.008)**	0.036 (0.006)	0.027 (0.006)	0.034 (0.006)	0.031 (0.006)	0.022 (0.008)	0.011 (0.004)
	A+ AD (N=16)	-0.010 (0.010)	0.009 (0.014)	0.039 (0.014)	0.064 (0.015)	0.048 (0.013)	0.005 (0.010)	0.049 (0.012)	0.038 (0.013)	0.048 (0.013)	0.039 (0.012)	0.061 (0.011)	0.002 (0.011)	0.030 (0.010)	0.035 (0.011)	0.045 (0.012)	0.020 (0.008)
**MRI-Free**	A- CU (N=43)	0.000 (0.003)	0.005 (0.004)	0.008 (0.007)	0.007 (0.007)	0.007 (0.006)	0.007 (0.004)	0.005 (0.006)	0.008 (0.006)	0.004 (0.007)	0.001 (0.006)	0.005 (0.005)	0.010 (0.004)	0.007 (0.005)	0.005 (0.006)	0.003 (0.006)	0.000 (0.003)
	A+ CU (N=45)	0.007 (0.003)	0.018 (0.005)	0.017 (0.007)	0.031 (0.007)	0.026 (0.006)	0.021 (0.004)	0.021 (0.006)	0.015 (0.006)	0.016 (0.006)	0.009 (0.006)	0.012 (0.005)	0.024 (0.004)	0.017 (0.005)	0.019 (0.006)	0.012 (0.006)	**0.007 (0.003)**
	A- MCI (N=23)	-0.006 (0.005)	0.002 (0.007)	0.006 (0.010)	0.012 (0.011)	0.008 (0.009)	-0.002 (0.006)	0.006 (0.009)	0.004 (0.010)	0.014 (0.010)	0.005 (0.009)	0.011 (0.007)	-0.001 (0.007)	0.004 (0.008)	0.005 (0.008)	0.008 (0.009)	0.003 (0.005)
	A+ MCI (N=40)	-0.004 (0.004)	0.021 (0.006)	0.027 (0.008)	0.057 (0.009)	0.047 (0.007)	0.024 (0.005)	0.039 (0.007)	0.026 (0.008)	0.028 (0.008)	**0.013 (0.007)**	0.031 (0.006)	0.020 (0.005)	0.037 (0.006)	0.034 (0.007)	0.021 (0.007)	0.010 (0.004)
	A+ AD (N=16)	-0.023 (0.009)	0.000 (0.012)	0.034 (0.013)	0.060 (0.014)	0.047 (0.012)	0.004 (0.009)	0.042 (0.011)	0.035 (0.012)	0.033 (0.012)	**0.023 (0.012)**	0.043 (0.009)	-0.002 (0.011)	0.033 (0.010)	0.036 (0.011)	0.029 (0.011)	0.009 (0.008)

P<0.001 P<0.01 P<0.05 **P<0.10**

S. Table 7: Impact of pipeline on annual change (top) and R^2 association between MRI-free and MRI-based slopes (bottom). A Pipeline x Time interaction term was included in linear mixed effects regression models to determine whether pipeline influenced rates of change in tau SUVR.

	Early Medial Temporal Lobe ROIs					Composite Cortical ROIs						Braak Stage-Based ROIs				
	**Hipp.**	**Amyg.**	**Precuneus**	**Inf. Temp.**	**Meta**	**MT**	**LT**	**MP**	**LP**	**F**	**O**	**B1/B2**	**B3**	**B4**	**B5**	**B6**
Pipeline differences Beta (SE)	0.001 (0.002)	0.001 (0.002)	0.001 (0.002)	0.001 (0.002)	0.001 (0.002)	0.001 (0.002)	0.000 (0.002)	0.001 (0.002)	0.000 (0.002)	-0.001 (0.002)	0.002 (0.002)	0.001 (0.003)	**0.003 (0.002)**	0.000 (0.002)	-0.001 (0.002)	0.001 (0.001)
MRI-Free vs MRI-Based R^2	0.80	0.88	0.95	0.94	0.96	0.85	0.96	0.94	0.92	0.96	0.84	0.46	0.95	0.97	0.96	0.89

P<0.001 P<0.01 P<0.05 **P<0.10**

S. Table 8: Longitudinal annual change differences in tau PET SUVR between diagnostic groups.

		Early Medial Temporal Lobe ROIs					Composite Cortical ROIs						Braak Stage-Based ROIs				
	**Group**	**Hipp.**	**Amyg.**	**Precuneus**	**Inf. Temp.**	**Meta**	**MT**	**LT**	**MP**	**LP**	**F**	**O**	**B1/B2**	**B3**	**B4**	**B5**	**B6**
**MRI-Based**	A- CU vs A+ CU	0.011 (0.005)	0.017 (0.008)	0.011 (0.011)	0.023 (0.011)	0.019 (0.009)	0.018 (0.006)	**0.017 (0.010)**	0.010 (0.010)	0.008 (0.010)	0.009 (0.010)	0.002 (0.007)	0.018 (0.007)	**0.014 (0.008)**	**0.014 (0.008)**	0.008 (0.009)	0.005 (0.006)
	A+ CU vs A+ MCI	-0.012 (0.006)	-0.007 (0.009)	0.013 (0.011)	0.026 (0.012)	0.021 (0.010)	0.002 (0.007)	0.017 (0.010)	0.011 (0.011)	0.016 (0.011)	0.005 (0.010)	0.027 (0.008)	0.001 (0.008)	0.017 (0.008)	0.013 (0.009)	0.010 (0.010)	0.006 (0.006)
	A+ MCI vs A- MCI	0.004 (0.008)	0.013 (0.012)	0.021 (0.014)	0.044 (0.015)	0.038 (0.013)	0.023 (0.009)	0.033 (0.013)	0.020 (0.014)	0.023 (0.014)	0.009 (0.013)	0.028 (0.010)	0.024 (0.009)	0.030 (0.010)	0.027 (0.011)	0.015 (0.013)	0.012 (0.008)
	A+ MCI vs A+ AD	-0.007 (0.011)	-0.004 (0.015)	0.013 (0.016)	0.008 (0.017)	0.003 (0.015)	**-0.019 (0.012)**	0.011 (0.015)	0.013 (0.016)	0.018 (0.016)	**0.025 (0.015)**	0.025 (0.012)	-0.025 (0.013)	-0.004 (0.012)	0.004 (0.012)	0.023 (0.014)	0.008 (0.009)
**MRI-Free**	A- CU vs A+ CU	0.007 (0.005)	0.013 (0.006)	0.008 (0.009)	0.024 (0.010)	0.018 (0.009)	0.014 (0.005)	**0.016 (0.008)**	0.007 (0.009)	0.011 (0.009)	0.009 (0.009)	0.007 (0.007)	0.015 (0.006)	0.010 (0.007)	**0.014 (0.008)**	0.009 (0.008)	0.007 (0.005)
	A+ CU vs A+ MCI	-0.011 (0.005)	0.003 (0.008)	0.011 (0.010)	0.027 (0.011)	0.021 (0.010)	0.003 (0.006)	0.018 (0.009)	0.011 (0.010)	0.012 (0.010)	0.003 (0.009)	0.019 (0.007)	-0.005 (0.007)	0.019 (0.008)	**0.015 (0.009)**	0.009 (0.009)	0.004 (0.006)
	A+ MCI vs A- MCI	0.002 (0.007)	0.019 (0.009)	**0.022 (0.013)**	0.045 (0.014)	0.038 (0.012)	0.026 (0.008)	0.032 (0.011)	**0.022 (0.012)**	0.014 (0.012)	0.008 (0.012)	0.020 (0.009)	0.021 (0.009)	0.032 (0.010)	0.029 (0.011)	0.013 (0.011)	0.008 (0.007)
	A+ MCI vs A+ AD	-0.019 (0.010)	-0.021 (0.013)	0.007 (0.015)	0.002 (0.017)	0.000 (0.014)	**-0.020 (0.010)**	0.003 (0.013)	0.008 (0.014)	0.006 (0.015)	0.010 (0.014)	0.012 (0.011)	**-0.022 (0.012)**	-0.003 (0.011)	0.02 (0.013)	0.008 (0.013)	-0.002 (0.009)

P<0.001 P<0.01 P<0.05 **P<0.10**

S. Table 8A: Raw vs. FDR-corrected P-values from S. Table 8 illustrating high consistency across pipelines, but diminished significance of subtle effects between CU groups.

Raw P-values

		Early Medial Temporal Lobe ROIs					Composite Cortical ROIs						Braak Stage-Based ROIs				
	**Group**	**Hipp.**	**Amyg.**	**Precuneus**	**Inf. Temp.**	**Meta**	**MT**	**LT**	**MP**	**LP**	**F**	**O**	**B1/B2**	**B3**	**B4**	**B5**	**B6**
**MRI-Based**	A- CU vs A+ CU	0.036	0.036	0.311	0.034	0.047	0.005	**0.089**	0.314	0.423	0.342	0.804	0.007	**0.059**	**0.078**	0.369	0.367
**MRI-Free**	A- CU vs A+ CU	0.124	0.042	0.374	0.018	0.039	0.011	**0.051**	0.433	0.217	0.304	0.298	0.014	0.141	**0.072**	0.259	0.178

Corrected P-values

		Early Medial Temporal Lobe ROIs					Composite Cortical ROIs						Braak Stage-Based ROIs				
	**Group**	**Hipp.**	**Amyg.**	**Precuneus**	**Inf. Temp.**	**Meta**	**MT**	**LT**	**MP**	**LP**	**F**	**O**	**B1/B2**	**B3**	**B4**	**B5**	**B6**
**MRI-Based**	A- CU vs A+ CU	**0.059**	**0.059**	0.311	**0.059**	**0.059**	0.030	0.267	0.508	0.508	0.508	0.804	0.035	0.130	0.130	0.259	0.223
**MRI-Free**	A- CU vs A+ CU	0.155	**0.070**	0.374	0.070	0.070	**0.067**	0.153	0.433	0.365	0.365	0.365	**0.070**	0.223	0.180	0.259	0.223

S. References:

Klunk, W.E., Koeppe, R.A., Price, J.C., Benzinger, T.L., Devous Sr, M.D., Jagust, W.J., Johnson, K.A., Mathis, C.A., Minhas, D., Pontecorvo, M.J., 2015. The Centiloid Project: standardizing quantitative amyloid plaque estimation by PET. Alzheimer's & dementia 11, 1–15.

Landau, S.M., Ward, T.J., Murphy, A., Iaccarino, L., Harrison, T.M., La Joie, R., Baker, S., Koeppe, R.A., Jagust, W.J., Initiative, A.D.N., 2023. Quantification of amyloid beta and tau PET without a structural MRI. Alzheimer's & Dementia 19, 444–455.

Murphy, A,E,, Lee, J., Chadwick, T., Ward, T.J., Baker, S., Landau, S., Harrison, T.M., Jagust, W., 2023. SCAN Amyloid PET MRI-free Processing. https://files.alz.washington.edu/scan/UCBerkeley_SCAN_Amyloid_MRIfree_Methods_20230818.pdf

Navitsky, M., Joshi, A.D., Kennedy, I., Klunk, W.E., Rowe, C.C., Wong, D.F., Pontecorvo, M.J., Mintun, M.A., Devous, M.D., 2018. Standardization of amyloid quantitation with florbetapir standardized uptake value ratios to the Centiloid scale. Alzheimer's and Dementia 14, 1565–1571. https://doi.org/10.1016/j.jalz.2018.06.1353

Rowe, C.C., Doré, V., Jones, G., Baxendale, D., Mulligan, R.S., Bullich, S., Stephens, A.W., De Santi, S., Masters, C.L., Dinkelborg, L., 2017. 18 F-Florbetaben PET beta-amyloid binding expressed in Centiloids. European journal of nuclear medicine and molecular imaging 44, 2053–2059.
